# Bayesian Hierarchical Models With Calibrated Mixtures of g‐priors for Assessing Treatment Effect Moderation in Meta‐Analysis

**DOI:** 10.1002/sim.70510

**Published:** 2026-04-02

**Authors:** Qiao Wang, Hwanhee Hong

**Affiliations:** ^1^ Department of Public Health East Carolina University Brody School of Medicine Greenville North Carolina USA; ^2^ Department of Biostatistics and Bioinformatics Duke University School of Medicine Durham North Carolina USA

**Keywords:** calibrated mixtures of g‐priors, individual participant‐level data, major depressive disorder, meta‐analysis, shrinkage method, treatment effect moderation

## Abstract

Assessing treatment effect moderation is critical in biomedical science and many other fields, as it guides personalized interventions to improve individual health outcomes. Individual participant‐level data meta‐analysis (IPD‐MA) offers a robust framework for such assessments by leveraging data from multiple studies. However, its performance is often compromised by real‐world challenges, including but not limited to high between‐study variability or small magnitude of moderation effect. Traditional Bayesian shrinkage methods have gained popularity in addressing these challenges, but are less suitable in MA, as their priors do not discern heterogeneous studies. In this paper, we propose calibrated mixtures of g‐priors in IPD‐MA to enhance efficiency and reduce risks in estimating moderation effects, providing a novel series of priors tailored for multiple studies by incorporating a study‐level calibration parameter and a moderator‐level shrinkage. This design offers a flexible range of shrinkage levels, allowing practitioners to evaluate moderator importance from conservative and optimistic perspectives. Compared with existing Bayesian shrinkage methods, our simulation studies demonstrate that calibrated mixtures of g‐priors exhibit equivalent or superior performances in estimating moderation effects. The benefits of the proposed methods are particularly pronounced in scenarios with high between‐study variability, high model sparsity, weak moderation effects, and correlated design matrices. We illustrate their application in assessing moderators of two treatments for major depressive disorder, using IPD from four randomized controlled trials.

## 1 Introduction

Evaluation of treatment effect moderation is essential in many fields, including epidemiology and biomedical science, as it helps measure differential responses to interventions and thus informs personalized intervention strategies [1, 2, 3]. However, investigating effect moderators using a single study often suffers from limited statistical power in detection and reduced efficiency in estimation [4, 5]. For example, in clinical trials, the standard recruitment strategy, which is designed to ensure sufficient power to detect the main effects, typically results in low power to identify the differential intervention effects, further reduced by the necessary corrections for multiple comparisons [6].

Individual participant data meta‐analysis (IPD‐MA) emerges as a robust method for synthesizing findings from all available relevant studies. Leveraging participant‐level data from multiple studies and properly accounting for between‐study variability, IPD‐MA not only enhances statistical power but also advances methods for evaluating effect moderation by increasing sample size and incorporating a participant's specific characteristics [7, 8, 9, 10, 11]. However, the application of IPD‐MA in assessing effect moderation is often hampered by real‐world challenges, such as weak signals of the effect moderators and the large number of potential effect moderators [12, 13, 14]. To address these challenges, several methods have been proposed to provide shrinkage estimators that improve estimation risk measures, defined as the expected loss of an estimator under a given decision rule. When we adopt the quadratic loss function as a decision rule, the risk measure is the mean squared error. These methods sacrifice bias to improve efficiency (i.e., bias‐variance tradeoff), mitigate estimation or prediction risk, and control model overfitting [13, 15, 16, 17, 18].

Among the proposed methods, Bayesian shrinkage methods, such as the Bayesian least absolute shrinkage and selection operator (LASSO) [19], the horseshoe prior method [20], and the stochastic search variable selection (SSVS) method [21], offer significant advantages in evaluating effect moderation due to their inherent flexibility in the prior specification and their capacity for direct uncertainty quantification. Specifically, the Bayesian LASSO heavily shrinks the coefficients toward zero by placing a Laplacian prior, while the horseshoe and SSVS methods are less restrictive, allowing the coefficients that represent true effects or signals in the data to avoid excessive penalization. These methods are straightforward and easily applicable when analyzing a single study. However, they might be less efficient for leveraging multiple studies in IPD‐MA as they do not adequately account for varying levels of variability or shrinkage across studies.

To overcome these limitations, mixtures of g‐priors offer a more flexible framework to leverage multiple studies due to its special covariance structure. Mixtures of g‐priors have been proposed as a Bayesian method for variable selection in a single study [22, 23, 24]. In multiple linear regression, Y=Xβ+ϵ, with $Y\in {\mathbb{R}}^{n},X\in {\mathbb{R}}^{n\times p},\beta \in {\mathbb{R}}^{p},$ and $\epsilon ∼N(0,\sigma^{2}I_{n})$, the g‐prior for β is specified as:

(1) $$\begin{array}{l} \beta |g,\sigma^{2}∼N_{p}(0,g\sigma^{2}{\left(X^{\prime }X\right)}^{-1}). \end{array}$$

When g is prespecified or fixed, known as Zellner's g‐prior, the posterior mean of β conditional on g is $g/(1+g){\left(X^{\prime }X\right)}^{-1}X^{\prime }Y$. This is the product of the shrinkage factor, g/(1+g), and the unbiased least squares estimator of β, ${\left(X^{\prime }X\right)}^{-1}X^{\prime }Y$. The shrinkage factor reflects the extent to which the posterior mean is pulled toward zero. When g follows a distribution, this defines the mixtures of g‐priors. In this case, the posterior mean of β is $E\left(g/(1+g)|Y\right){\left(X^{\prime }X\right)}^{-1}X^{\prime }Y$, where E(g/(1+g)|Y) is the shrinkage factor, quantifying the shift in mean relative to the least squares estimator. Mixtures of g‐priors offer several advantages: they provide a proper conjugate prior [25], establish strong theoretical foundations for model selection, and enhance computational efficiency. However, the properties of the resulting shrinkage estimators have been rarely explored, especially in multiple studies [26, 27, 28, 29].

In this paper, we focus on estimation rather than detection of moderation effects, and propose the calibrated mixtures of g‐priors in IPD‐MA to assess treatment effect moderation by effectively leveraging multiple studies. The proposed priors are a novel shrinkage prior tailored for multiple studies, incorporating both study‐level and moderator‐level information to improve the estimation of moderation effects with greater efficiency and lower risk measures compared to standard Bayesian shrinkage methods. The proposed calibrated mixtures of g‐priors allow different functions to integrate study‐level information and offer a wide spectrum of shrinkage levels across moderators, providing a comprehensive assessment of risk measures from conservative to optimistic approaches.

The remainder of this paper is presented as follows. Section 2 introduces the IPD‐MA model, Section 3 reviews widely used standard Bayesian shrinkage methods, and Section 4 introduces our naïve and calibrated mixtures of g‐priors. In Section 5, we evaluate the performance of our proposed methods compared to standard methods through an extensive simulation study under various settings. In Section 6, we apply these methods to a real data example of IPD‐MA, combining four randomized controlled trials (RCTs) comparing two psychiatric treatments for patients with major depressive disorder. Finally, Section 7 concludes with a discussion of results, provides recommendations for practitioners, and suggests future research directions.

## 2 Linear IPD‐MA Model

IPD‐MA is a powerful approach that synthesizes participant‐level data from multiple studies in estimating treatment effects. It enables personalized medical decisions by incorporating a participant's specific characteristics and assessing effect moderators through modeling covariate‐treatment interactions. When specifying an IPD‐MA model, random effects are commonly included to model variability across studies, as these studies are often conducted by different research teams across diverse healthcare systems, which can lead to variations in participant management practices and health outcomes. In addition, we consider a one‐stage IPD‐MA model rather than a two‐stage approach in this work, and interested readers may refer to [9, 11, 30] for comparisons of one‐stage and two‐stage approaches.

Without loss of generality, we assume that $y_{ij}$ is the continuous outcome for the jth participant in the ith study, where i=1,2,⋯,I, $j=1,2,\cdots ,n_{i}$, and $n_{i}$ is the sample size for the ith study. The IPD‐MA can then be fitted using the following linear mixed effects regression model:

(2) $$\begin{array}{ll} y_{ij} & =\mu +t_{ij}\alpha +x_{ij}^{\prime }\beta +x_{ij}^{em^{\prime }}\gamma +u_{\mu i}+t_{ij}u_{\alpha i}+x_{ij}^{em^{\prime }}u_{i}+\epsilon_{ij},\\ u_{\mu i} & ∼N(0,\tau_{\mu }^{2}),u_{\alpha i}∼N(0,\tau_{\alpha }^{2}),u_{ki}∼N(0,\tau_{k}^{2})\;\text{for}\;u_{ki}\in u_{i},\; \end{array}$$

where μ is the intercept, α is the conditional treatment effect with the treatment indicator $t_{ij}$ (1 for the treatment and 0 for the control group), $\beta ={\left(\beta_{1},\cdots ,\beta_{p}\right)}^{\prime }\in {\mathbb{R}}^{p}$ is a set of coefficients for the centered baseline covariates 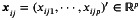, $\gamma ={\left(\gamma_{1},\cdots ,\gamma_{d}\right)}^{\prime }\in {\mathbb{R}}^{d}$ represents the coefficients for the interactions between the treatment and some baseline characteristics $x_{ij}^{em}={\left(t_{ij}x_{ij1},\cdots ,t_{ij}x_{ijd}\right)}^{\prime }\in {\mathbb{R}}^{d}$, where 0≤d≤p. Here, γ is the key parameter. We refer to γ as moderation effects, and baseline characteristics as moderators or effect moderators. For example, age could be a moderator, and the moderation effect is then described by the age‐by‐treatment interaction. $u_{\mu i}$ and $u_{\alpha i}$ are the random effects for the intercept and the conditional treatment effect, with $\tau_{\mu }^{2}\;\text{and}\;\tau_{\alpha }^{2}$ capturing the between‐study variability, respectively. $u_{i}={\left(u_{1i},\cdots ,u_{ki},\cdots ,u_{di}\right)}^{\prime }\in {\mathbb{R}}^{d}$ is a vector of random effects for the moderation effects, where $u_{ki}$ models the random effects of the $k^{th}$ effect moderator with $\tau_{k}^{2}$ capturing its between‐study variability. Finally, $\epsilon_{ij}∼N(0,\sigma_{i}^{2})$ is the random noise for study i. Although it is also feasible to specify random effects for the baseline effects β, we consider them fixed for simplicity. The parameter of interest is the magnitude of the moderation effect, γ. In practice, the random effects $u_{i}$ for moderation effects can be excluded to reduce the model complexity. The IPD‐MA model in (2) assumes that the common fixed moderation effects γ shared across studies, and allows study‐specific deviations $u_{ki}$ around the common fixed moderation effects.

We assess moderation effects by comparing estimation metrics of γ under various prior specifications. To ensure a fair comparison, we employ the same priors for the rest of the parameters across methods. Specifically, we adopt a noninformative prior, $\pi (\mu ,\alpha ,\beta_{,}\sigma^{2})\propto 1/\sigma^{2}$ for coefficients other than γ. For the parameters of variability between studies, we use $\tau_{\mu },\tau_{\alpha },\tau_{k}∼C^{+}(0,1)$, where $C^{+}(0,1)$ is a half Cauchy distribution [31]. We acknowledge that choosing prior distributions for $\tau_{\mu },\tau_{\alpha },\;\text{and}\;\tau_{k}$ is an important task in MA, but it falls outside the scope of this paper as our primary goal is to propose a prior for moderation effects in multiple studies; however, our simulation studies (not shown) indicate that varying these priors will have minimal impact on the relative performances of the methods considered in this work. In the following subsections, we review existing Bayesian shrinkage methods and introduce our naïve and calibrated mixtures of g‐priors methods. In addition to these shrinkage priors, we consider the classical Flat method, which is defined by π(γ)∝1. Table 1 summarizes all Bayesian methods considered in this paper.

**TABLE 1:** Summary of methods.

Methods	Prior distribution for $\gamma_{k}$	Hyperparameter specification
**Non‐shrinkage method**		
Flat	$\gamma_{k}\propto 1$	
**Two existing shrinkage methods**		
HS	$\gamma_{k}|\lambda_{k},\tau ∼N(0,\lambda_{k}^{2}\tau^{2})$	$\lambda_{k}∼C^{+}(0,1),\tau ∼C^{+}(0,1)$
SSVS	$\gamma_{k}|I_{k},h_{k},\eta ,∼(1-I_{k})N(0,\eta^{2})+I_{k}N(0,h_{k}\eta^{2})$	$I_{k}∼Ber(0.5),\eta ∼Uniform(0,c)$ with small c
**NMG methods**		
UIP	$\gamma_{k}|g_{k},\Lambda ∼N\left(0,g_{k}{\left(x_{k}^{\prime }\Lambda x_{k}\right)}^{-1}\right)$	$g_{k}=N$
ZS	$\gamma_{k}|g_{k},\Lambda ∼N\left(0,g_{k}{\left(x_{k}^{\prime }\Lambda x_{k}\right)}^{-1}\right)$	$g_{k}|N∼IG\left(1/2,N/2\right)$
HG (a=3)	$\gamma_{k}|g_{k},\Lambda ∼N\left(0,g_{k}{\left(x_{k}^{\prime }\Lambda x_{k}\right)}^{-1}\right)$	$g_{k}/(g_{k}+1)∼Beta(1,3/2-1)$
HG (a=4)	$\gamma_{k}|g_{k},\Lambda ∼N\left(0,g_{k}{\left(x_{k}^{\prime }\Lambda x_{k}\right)}^{-1}\right)$	$g_{k}/(g_{k}+1)∼Beta(1,4/2-1)$
HGN (a=3)	$\gamma_{k}|g_{k},\Lambda ∼N\left(0,g_{k}{\left(x_{k}^{\prime }\Lambda x_{k}\right)}^{-1}\right)$	$g_{k}/(g_{k}+N)∼Beta(1,3/2-1)$
HGN (a=4)	$\gamma_{k}|g_{k},\Lambda ∼N\left(0,g_{k}{\left(x_{k}^{\prime }\Lambda x_{k}\right)}^{-1}\right)$	$g_{k}/(g_{k}+N)∼Beta(1,4/2-1)$
**CMG methods**		
CUIP	$\gamma_{k}|g_{k},\Lambda ∼N\left(0,g_{k}{\left(x_{k}^{\prime }\Lambda^{⋆}x_{k}\right)}^{-1}\right)$	$g_{k}=1$, $f(n_{i})=\sqrt{N}$
CZS	$\gamma_{k}|g_{k},\Lambda^{⋆}∼N\left(0,g_{k}{\left(x_{k}^{\prime }\Lambda^{⋆}x_{k}\right)}^{-1}\right)$	$g_{k}∼IG\left(1/2,1/2\right),\;f(n_{i})=n_{i}p_{i},\;p_{i}∼Uniform(1/n_{i},1)$
CMG‐S1‐n	$\gamma_{k}|g_{k},\Lambda^{⋆}∼N\left(0,g_{k}{\left(x_{k}^{\prime }\Lambda^{⋆}x_{k}\right)}^{-1}\right)$	$g_{k}/(g_{k}+1)∼Beta(2,b_{i}),\;f(n_{i})=n_{i}p_{i},\;b_{i}∼Uniform(0,2),\;p_{i}∼Uniform(1/n_{i},1)$
CMG‐S2‐n	$\gamma_{k}|g_{k},\Lambda^{⋆}∼N\left(0,g_{k}{\left(x_{k}^{\prime }\Lambda^{⋆}x_{k}\right)}^{-1}\right)$	$g_{k}/(g_{k}+1)∼Beta(1,1),\;f(n_{i})=n_{i}p_{i},\;p_{i}∼Uniform(1/n_{i},1)$
CMG‐S3‐n	$\gamma_{k}|g_{k},\Lambda^{⋆}∼N\left(0,g_{k}{\left(x_{k}^{\prime }\Lambda^{⋆}x_{k}\right)}^{-1}\right)$	$g_{k}/(g_{k}+1)∼Beta(b_{i},2),\;f(n_{i})=n_{i}p_{i},\;b_{i}∼Uniform(0,2),\;p_{i}∼Uniform(1/n_{i},1)$
CMG‐S1‐log	$\gamma_{k}|g_{k},\Lambda^{⋆}∼N\left(0,g_{k}{\left(x_{k}^{\prime }\Lambda^{⋆}x_{k}\right)}^{-1}\right)$	$g_{k}/(g_{k}+1)∼Beta(2,b_{i}),\;f(n_{i})=log(n_{i}p_{i}),\;b_{i}∼Uniform(0,2),\;p_{i}∼Uniform(1/n_{i},1)$
CMG‐S2‐log	$\gamma_{k}|g_{k},\Lambda^{⋆}∼N\left(0,g_{k}{\left(x_{k}^{\prime }\Lambda^{⋆}x_{k}\right)}^{-1}\right)$	$g_{k}/(g_{k}+1)∼Beta(1,1),\;f(n_{i})=log(n_{i}p_{i}),\;p_{i}∼Uniform(1/n_{i},1)$
CMG‐S3‐log	$\gamma_{k}|g_{k},\Lambda^{⋆}∼N\left(0,g_{k}{\left(x_{k}^{\prime }\Lambda^{⋆}x_{k}\right)}^{-1}\right)$	$g_{k}/(g_{k}+1)∼Beta(b_{i},2),\;f(n_{i})=log(n_{i}p_{i}),\;b_{i}∼Uniform(0,2),\;p_{i}∼Uniform(1/n_{i},1)$
CMG‐S1‐pow	$\gamma_{k}|g_{k},\Lambda^{⋆}∼N\left(0,g_{k}{\left(x_{k}^{\prime }\Lambda^{⋆}x_{k}\right)}^{-1}\right)$	$g_{k}/(g_{k}+1)∼Beta(2,b_{i}),\;f(n_{i})=n_{i}^{p_{i}},\;b_{i}∼Uniform(0,2),\;p_{i}∼Uniform(0,1)$
CMG‐S2‐pow	$\gamma_{k}|g_{k},\Lambda^{⋆}∼N\left(0,g_{k}{\left(x_{k}^{\prime }\Lambda^{⋆}x_{k}\right)}^{-1}\right)$	$g_{k}/(g_{k}+1)∼Beta(1,1),\;f(n_{i})=n_{i}^{p_{i}},\;p_{i}∼Uniform(0,1)$
CMG‐S3‐pow	$\gamma_{k}|g_{k},\Lambda^{⋆}∼N\left(0,g_{k}{\left(x_{k}^{\prime }\Lambda^{⋆}x_{k}\right)}^{-1}\right)$	$g_{k}/(g_{k}+1)∼Beta(b_{i},2),\;f(n_{i})=n_{i}^{p_{i}},\;b_{i}∼Uniform(0,2),\;p_{i}∼Uniform(0,1)$

## 3 Two Existing Bayesian Shrinkage Methods

### 3.1 Horseshoe Prior

The classical horseshoe (HS) prior [20] and its variants [32, 33, 34, 35] are widely used for variable selection with high‐dimensional data. The classical horseshoe prior is defined as follows:

$$\begin{array}{l} \gamma_{k}|\lambda_{k},\tau ∼N(0,\lambda_{k}^{2}\tau^{2}),\;\lambda_{k}∼C^{+}(0,1),\;\tau ∼C^{+}(0,1), \end{array}$$

where $\lambda_{k}$ governs local shrinkage and τ controls global shrinkage. Thus, it differentiates the global and local shrinkage, facilitating the Bayesian estimation in a sparse model.

### 3.2 Stochastic Search Variable Selection

The stochastic search variable selection (SSVS) method is another popular Bayesian method for model selection in high‐dimensional data [18, 21, 36]. Unlike the horseshoe prior, which utilizes scale mixtures, SSVS comprises a mixture of two normal distributions. Although numerous variants of SSVS have been proposed for various applications, we employ the SSVS specified in [18] as follows:

$$\begin{array}{l} \gamma_{k}|I_{k},h_{k},\eta ∼(1-I_{k})N(0,\eta^{2})+I_{k}N(0,h_{k}\eta^{2}). \end{array}$$

The first component controls shrinkage by centering a point mass around zero with η∼Unif(0,c), where c is a small constant such as 5. The second component lessens the shrinkage for the true effect moderator with a large $h_{k}$, such as 100. Finally, $I_{k}∼Ber(0.5)$ serves as a binary indicator of the presence of a true effect moderator.

## 4 Naïve and Calibrated Mixtures of G‐priors in IPD‐MA

In this section, we propose two approaches: the naïve and the calibrated mixtures of g‐priors in IPD‐MA. The naïve approach accounts for study‐specific noises, and is a direct extension of the simple mixtures of g‐priors used in a single‐study setting. The calibrated approach refines this approach by incorporating additional study‐level information and different shrinkage levels.

### 4.1 Naïve Mixtures of g‐priors in IPD‐MA

In model (2), assume that $\sigma_{i}=\sigma$ for all studies, the simple mixtures of g‐priors in (1) are respecified as follows:

$$\gamma |g,\sigma^{2}∼N_{d}\left(0,g\sigma^{2}{\left(X^{em^{\prime }}X^{em}\right)}^{-1}\right),g>0,$$

where $X^{em}$ is the design matrix with the (i,j) element denoted by $x_{ij}^{em}$. The simple g‐prior has several limitations. First, it imposes uniform shrinkage across both true and false effect moderators via the parameter g, leading to excessive shrinkage of true effect moderators, while insufficiently shrinking coefficients of the non‐effect moderators. Second, the assumption that the noise parameters are the same in all studies (i.e., $\sigma_{i}$ = σ for any i) is overly stringent. Third, the requirement that $X^{em}$ has full column rank in order for ${\left(X^{em\prime }X^{em}\right)}^{-1}$ to exist is overly restrictive, particularly when dealing with a large number of potential effect moderators that are highly correlated.

To address these issues, we first propose the naïve mixtures of g‐priors (NMG) methods for $\gamma_{k}$ in model (2) as follows:

(3) $$\begin{array}{l} \gamma_{k}|g_{k},t,x_{\left[k\right]},\Lambda ∼N\left(0,g_{k}{\left[{\left(t\circ x_{\left[k\right]}\right)}^{\prime }\Lambda (t\circ x_{\left[k\right]})\right]}^{-1}\right),g_{k}>0, \end{array}$$

where $g_{k}$ is the shrinkage parameter for the $k^{th}$ potential effect moderator, ∘ is the Hadamard product, 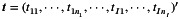 is the vector of treatment indicators for all participants across I studies, $x_{\left[k\right]}={\left(x_{11k},\cdots ,x_{1n_{1}k},\cdots ,x_{I1k},\cdots ,x_{In_{I}k}\right)}^{\prime }$ is the vector of covariates for the $k^{th}$ effect moderator, $\Lambda =diag(1/\sigma_{1}^{2}1_{n_{1}},\cdots ,1/\sigma_{I}^{2}1_{n_{I}})$ scaling $t\circ x_{\left[k\right]}$ according to each study's specific $\sigma_{i}^{2}$. The prior in (3) for a single study is commonly referred to as “independent mixtures of g‐priors” or “block mixtures of g‐priors” [28, 37, 38]. We consider several specifications for $g_{k}$ in the NMG methods.

#### 4.1.1 Unit Information Prior in NMG

The unit information prior (UIP) prespecifies $g_{k}$ based on the total sample size of all studies:

$$g_{k}=N=\overset{I}{\underset{i=1}{\sum }}n_{i}.$$

This represents the prior's contribution equivalent to one observation's worth of information [39].

#### 4.1.2 Zellner‐Siow Prior in NMG

The Zellner‐Siow (ZS) prior [22] utilizes an inverse‐Gamma (IG) distribution for $g_{k}$, incorporating the total sample size into its scale parameter. The ZS prior in IPD‐MA is defined as:

$$g_{k}|N∼IG\left(1/2,N/2\right).$$

Integrating $g_{k}$ from (3) results in $\gamma_{k}|t,x_{\left[k\right]},\Lambda$ following a Cauchy distribution. Therefore, the ZS prior together with (3) is the hierarchical representation of $\gamma_{k}|t,x_{\left[k\right]},\Lambda$, providing computational efficiency.

#### 4.1.3 Hyper‐g Prior in NMG

The hyper‐g (HG) prior, proposed by [24], specifies a hyperprior to $g_{k}$ as follows:

$$\pi (g_{k}|a)=\frac{\left(a-2\right)}{2}{\left(1+g_{k}\right)}^{-a/2}.$$

This implies that $g_{k}/(1+g_{k})∼Beta(1,a/2-1)$, where Beta(r,s) denotes a Beta distribution with shape parameter r and scale parameter s. Different values of a lead to various prior shrinkage levels for $\gamma_{k}$. Commonly used values for a are 3 and 4; a=3 places more mass of the shrinkage factor $g_{k}/(1+g_{k})$ around 1, while a=4 results in a uniform prior to $g_{k}/(1+g_{k})$.

#### 4.1.4 Hyper‐g/N Prior in NMG

The hyper‐g/N (HGN) prior, proposed by [24], is an extended version of the HG prior. It adapts the level of prior influence based on the number of observations, and it is specified as:

$$\pi (g_{k}|a,N)=\frac{\left(a-2\right)}{2N}{\left(1+\frac{g_{k}}{N}\right)}^{-a/2},$$

which implies that $g_{k}/(g_{k}+N)∼Beta(1,a/2-1)$. Incorporating the sample size N in the formula addresses the model selection inconsistency observed in the HG prior. That is, the HG prior fails to guarantee posterior probabilities converging to 1 for the true model as the sample size increases. Similarly, we consider a=3 and a=4 for the HGN prior.

#### 4.1.5 Limitations of NMG

The NMG methods offer a straightforward framework for applying mixtures of g‐priors when combining multiple studies in IPD‐MA. However, they still present two key limitations. First, the HG prior applies the Beta(1,a/2−1) distribution to $g_{k}/(1+g_{k})$, where the distribution increases monotonically for 2<a<4 and decreases for a>4. Although choosing 2<a<4 ensures a proper prior and mitigates excessive shrinkage, this monotonicity causes the shrinkage factor's distribution to be skewed heavily towards 1 or 0. While this may be beneficial for model selection, it is less suitable for parameter estimation. To address this, a heavy‐tailed hyperprior distribution for $g_{k}/(1+g_{k})$ needs to be studied to allow for more flexible shrinkage.

Second, the UIP, ZS, and HGN priors rely on the total number of observations N from all studies in the IPD‐MA. While including N in the Bayesian model selection is common with two typical examples of the Bayesian Information Criterion (BIC) and the ZS prior, [40] and [41] have proposed to use the effective sample size (TESS) instead of a simple number of observations to achieve model selection consistency. Drawing inspirations from the TESS, it becomes apparent that using N in the prior assumes that each participant contributes equally to the estimation of $\gamma_{k}$, overlooking differences in individuals across studies and imposing insufficient shrinkage of the estimates. Thus, alternative approaches that better leverage the total number of observations in the hyperprior for $\gamma_{k}$ are needed to improve the estimation of moderation effects. However, the formal derivation of TESS is designed for the model selection and remains complex even with a single study, we adopt a more data‐driven approach, described in the following section, to calibrate the sample size contribution for each study.

### 4.2 Calibrated Mixtures of g‐priors in IPD‐MA

To address the aforementioned limitations of the NMG methods, we propose the calibrated mixtures of g‐priors (CMG) methods in IPD‐MA as follows:

(4) $$\begin{array}{ll} \gamma_{k}|g_{k},\Lambda^{⋆},t,x_{\left[k\right]} & ∼N\left(0,g_{k}{\left[{\left(t\circ x_{\left[k\right]}\right)}^{\prime }\Lambda^{⋆}(t\circ x_{\left[k\right]})\right]}^{-1}\right),g_{k}>0,\\ \Lambda^{⋆} & =diag\left(\sigma_{1}^{-2}f{\left(n_{1}\right)}^{-1}1_{n_{1}},\cdots ,\sigma_{I}^{-2}f{\left(n_{I}\right)}^{-1}1_{n_{I}}\right), \end{array}$$

where $f(n_{i})$ in $\Lambda^{⋆}$ is the study‐specific sample size tuning function, which scales the design matrix within each study. The CMG methods adjust different shrinkage levels across potential effect moderators through $g_{k}$, and control the expected contribution of observations to the estimation within each study through $f(n_{i})$. We consider 9 different prior specifications for the CMG methods, representing combinations of three hyperpriors for $g_{k}$ and three functions for $f(n_{i})$ (see Table 1).

#### 4.2.1 Hyperprior for $g_{k}$ in CMG

Motivated by the HG prior, three hyperpriors for $g_{k}$ to reflect different levels of prior shrinkage are defined as follows:

- Shrinkage 1 (S1):
$$\pi_{S_{1}}(g_{k}|b_{k})=\frac{g_{k}}{{\left(1+g_{k}\right)}^{b_{k}+2}B(2,b_{k})},b_{k}\leq 2,$$
  where $B(2,b_{k})$ is the Beta function. The S1 hyperprior is equivalent to $g_{k}/(1+g_{k})∼Beta(2,b_{k})$. Here, $b_{k}$ is used to control the skewness of $g_{k}/(1+g_{k})$, with $b_{k}$ either prespecified as a value less than 2 or assigned a hyperprior. For example, we assume $b_{k}∼Uniform(0,2)$, then the S1 hyperprior represents the least shrinkage, with a conceptually expected prior shrinkage of $E(g_{k}/(1+g_{k}))=ln(2)\approx 0.69$, indicating an average prior shrinkage of 31% toward 0 in coefficients.
- Shrinkage 2 (S2):
$$\pi_{S_{2}}(g_{k})=\frac{1}{{\left(1+g_{k}\right)}^{2}},$$
  which is equivalent to $g_{k}/(1+g_{k})∼Beta(1,1)$, implying a conceptually average prior shrinkage of 50% toward 0 in coefficients. This is the same as the HG prior in (4.1.3) with a=4 and $f(n_{i})=1$.
- Shrinkage 3 (S3):
$$\pi_{S_{3}}(g_{k}|b_{k})=\frac{g_{k}^{b_{k}-1}}{{\left(1+g_{k}\right)}^{b_{k}+2}B(b_{k},2)},b_{k}\leq 2,$$
  which is equivalent to $g_{k}/(1+g_{k})∼Beta(b_{k},2)$, where $b_{k}\leq 2$ ensures that $g_{k}/(1+g_{k})$ is right‐skewed. We follow the same rule as in S1 to specify $b_{k}$. If $b_{k}∼Uniform(0,2)$, S3 results in a conceptually expected prior shrinkage of 69% toward 0 in coefficients.

Overall, the S1 hyperprior results in the least shrinkage of point estimates of $\gamma_{k}$ toward 0, while S3 produces the most shrinkage when the same $f(n_{i})$ is used. When S3 is applied to all potential effect moderation, the resulting point estimates of $\gamma_{k}$ are the most conservative (i.e., close to the null value). This suggests that an effect of moderator with a relatively large estimated effect size under S3 may have a stronger influence compared to those with small effect sizes. We denote the CMG methods under each shrinkage level with the suffixes −S1, −S2, and −S3.

#### 4.2.2 Hyperprior for $f(n_{i})$ in CMG

The sample size tuning function $f(n_{i})$ for study i in the CMG methods is a function of the number of observations $n_{i}$. It is designed to calibrate how many observations are expected to contribute to the estimation of $\gamma_{k}$. Additionally, we impose the constraint $f(n_{i})\leq n_{i}$, ensuring that the contribution does not exceed the total number of observations in each study. This constraint can be implemented by including an unknown tuning parameter $p_{i}$ in $f(n_{i})$, and we consider three functions:

- n: $f(n_{i}|p_{i})=n_{i}p_{i},p_{i}∼\text{Uniform}(1/n_{i},\;1)$.
  This linear form grows fastest and thus induces the weakest shrinkage among the three functions. The tuning parameter $p_{i}$ adaptively modulates each study's contribution in the prior precision based on the observed data. From an information perspective, $p_{i}$ controls the number of observations expected to be leveraged, with at least one observation and up to $n_{i}$ observations.
- log: $f(n_{i}|p_{i})=\log(n_{i}p_{i}),p_{i}∼\text{Uniform}(1/n_{i},\;1)$.
  This logarithmic form yields the strongest shrinkage among the three functions. Similarly, $p_{i}$ adaptively distributes each study's contribution, ranging minimally from a single observation up to the full study size $n_{i}$ observations.
- pow: $f(n_{i}|p_{i})=n_{i}^{p_{i}},p_{i}∼\text{Uniform}(0,\;1)$.
  This fractional‐power function provides a middle‐ground between the linear and logarithmic forms. The tuning parameter $p_{i}$'s range reflects the number of observations expected to be leveraged, with at least one observation and up to $n_{i}$ observations.

The proposed study‐level sample size tuning function $f(n_{i})$ governs *study‐specific* shrinkage through the prior variance of moderation effects. The functional form of $f(n_{i})$ provides flexible specifications of growth rates and adaptively distributes study‐level contributions in the prior precision through the tuning parameter $p_{i}$, thereby yielding various shrinkage strengths and benefiting the estimation of moderation effects.

The specification of $f(n_{i})$ involves three important considerations. First, a sample‐size component is important in specifying calibrated mixtures of g‐prior. Without incorporating sample size in the prior (e.g., $f(n_{i})=1$), one large study can induce a large prior precision, leading to excessive shrinkage toward zero regardless of the true moderation effects. Second, given the necessity of including sample size, it is better to perform tuning to the sample size component, and a study‐level sample size tuning is encouraged. Using only the total sample size $f(n_{i})=N=\sum_{i}n_{i}$ without any tuning results in a diffuse prior with under‐shrinkage, as it merely rescales $g_{k}$ by a large constant. In contrast, the proposed study‐level tuning functions $f(n_{i}|p_{i})$ together with $p_{i}$ adaptively distribute each study's contribution to the prior precision based on the sample size across studies. Third, we restrict our considerations to basic functions that grow at most linearly to prevent overly diffused priors and insufficient shrinkage. Functions with different sublinear growth rates with different tuning parameters also result in distinct magnitudes of shrinkage. Admittedly, more complex and flexible specifications of $f(n_{i}|p_{i})$ are possible; however, we choose basic functions for clarity and computational efficiency. To further clarify the mechanism of the study‐level sample size tuning function $f(n_{i}|p_{i})$, we provide additional details in the Supporting Information Section S1. We denote the CMG methods under each of the study‐level tuning functions with the suffixes −n, −log, and −pow.

### 4.3 Other Specifications for CMG

In addition to the hyperpriors introduced in Section 4.2.1, other forms of $g_{k}$ and $f(n_{i})$ can be considered in the CMG methods. In this subsection, we introduce two additional forms of the CMG methods.

#### 4.3.1 Calibrated UIP

The calibrated UIP (CUIP) is defined by setting $g_{k}=1$ and $f(n_{i})=\sqrt{N}$ in the CMG methods. This simplifies the study‐level sample size calibration to an overall sample size adjustment, reducing the number of parameters and providing a simple calibration. A similar specification was studied in analyzing a single study [42].

#### 4.3.2 Calibrated ZS Prior

The extension of the ZS prior to the calibrated ZS prior (CZS) requires the representation of the ZS prior. Note that the ZS prior in (4.1.2) is equivalent to:

$$\begin{array}{ll} & \gamma_{k}|g_{k},N,\Lambda ,t,x_{\left[k\right]}\\ & \;∼N\left(0,g_{k}N{\left[{\left(t\circ x_{\left[k\right]}\right)}^{\prime }\Lambda (t\circ x_{\left[k\right]})\right]}^{-1}\right),g_{k}∼IG(1/2,1/2). \end{array}$$

The CZS is then defined by replacing Λ/N with $\Lambda^{⋆}$, and applying $g_{k}∼IG(1/2,1/2)$. For CZS, we only consider the sampling size tuning function $f(n_{i}|p_{i})=n_{i}p_{i}$ in $\Lambda^{⋆}$ with $p_{i}∼Uniform(1/n_{i},1)$ for simplicity.

### 4.4 Computation

Our proposed methods can be efficiently implemented using Markov Chain Monte Carlo (MCMC) via Just Another Gibbs Sampler (JAGS) within the R environment. For both simulation studies and data analyses, we use two MCMC chains, each with 20,000 samples. To improve the chain mixing properties, we apply thinning by retaining every $10^{th}$ sample and discard the first 10,000 samples as burn‐in. A sample R code to implement these methods is available from https://github.com/QWCodeShare/ModerationPaper.git.

## 5 Simulation

Our simulation study has three objectives. First, we compare the performance of 20 different methods listed in Table 1 in estimating moderation effects. Second, we study the bias‐variance tradeoff and participant‐specific estimation across all methods. Third, we discern specific settings where our proposed CMG methods offer better performance than other methods.

### 5.1 Simulation Setup

We generate IPD‐MA data containing five trials (I=5) of which the sample size $n_{i}$ varies from 100 to 150. We consider 8 baseline covariates (i.e., p=8) of which a subset is the true effect moderators. Continuous outcomes are simulated based on the model (2) with true parameters set to an intercept of μ=2, a conditional treatment effect of α=3, coefficients for baseline covariates of β=(1.8,2.7,2.3,1.5,1.7,2.2,1.3,2.6), noise $\left(\sigma_{1},\ldots ,\sigma_{5})=(3.5,2.5,2.1,2.8,3\right)$, between‐trial (i.e., between‐study) variability for the intercept of $\tau_{\mu }=1.5$, between‐trial variability for α of $\tau_{\alpha }=1.5$.

We vary true value settings for the remaining parameters of moderation effects, and use “EM” as the abbreviation of effect moderation to save space in all tables and figures.

a. We consider three settings for the between‐trial variability of the moderation effect $\gamma_{k}$. Different between‐trial variability for the $k^{th}$ effect moderator is set through $\tau_{k}$: $\tau_{k}\in (1.5,2.5)$ represents high variability (i.e., $\tau_{k}$ varies from 1.5 to 2.5), $\tau_{k}\in (0.5,1.5)$ is medium variability, and $\tau_{k}=0$ means no variability.
b. We consider three settings for the model sparsity. The model sparsity is determined by the number of true effect moderators, of which the corresponding coefficient elements in γ are nonzero. High sparsity corresponds to 2 out of 8 covariates being true effect moderators. Medium sparsity sets 4 out of 8 covariates as true effect moderators, and low sparsity sets 6 out of 8 covariates as true effect moderators.
c. We consider two settings for the correlation matrices when simulating baseline covariates $x_{ij}$ from a multivariate normal distribution (MVN) with $x_{ij}∼MVN(0,\sum_{i}^{B})$. Here, $\sum_{i}^{B}=\sigma_{is}^{B}\sigma_{it}^{B}\rho_{ist}^{B}$, with $\sigma_{is}^{B}=\sigma_{it}^{B}=1$ representing the standard deviations of baseline covariates s and t, and $\rho_{ist}^{B}$ representing the correlation among these characteristics. We consider no correlation (i.e., $\rho_{ist}^{B}=0$), and high correlation $0.5<\rho_{ist}^{B}<0.9$ for s≠t among baseline covariates.
d. We vary the strength of the true effect moderators. The first setting is strong effect moderators with $\gamma_{k}=1.5$ (i.e., 50% of the size of the conditional treatment effect, α=3). The second setting is weak effect moderators with $\gamma_{k}=0.75$ (i.e., 25% of the size of the conditional treatment effect, α=3).

As a result, we consider a total of 36 simulation settings. For each setting, we simulate 500 datasets and fit the 20 methods listed in Table 1. We additionally conducted simulation studies under a broader range of settings to assess the robustness of our findings. Specifically, we considered two settings for the number of studies (5 and 10), and three levels of sample sizes within each study: small ($n_{i}$ varies from 50 to 100), medium ($n_{i}$ varies from 100 to 150), and large ($n_{i}$ varies from 150 to 200), under the design of weak moderation effects, high sparsity of moderation effects, and high between‐trial variability and correlated covariates. These additional simulation settings do not substantially alter the relative performance of the proposed and competing methods in estimating moderation effects (Figure S4 in Supporting Information Section S2); subsequently, we present and interpret results primarily under the main simulation settings.

### 5.2 Simulation Performance Assessment

The relative performances of methods are evaluated by three metrics. First, for $\gamma \in {\mathbb{R}}^{d}$ (i.e., d=8), we calculate the average relative root mean squared error (ARRMSE) defined as:

(5) $$\begin{array}{ll} ARRMSE(\gamma ) & =\frac{\sqrt{\sum_{r=1}^{500}\sum_{k=1}^{d}{\left({\hat{\gamma }}_{k}^{\left(r\right)}-\gamma_{k}\right)}^{2}/500}}{d\sum_{k=1}^{d}|\gamma_{k}|}, \end{array}$$

where r denotes the number of datasets to calculate the metrics, k indexes the individual parameters within γ, and ${\hat{\gamma }}_{k}^{\left(r\right)}$ is the posterior mean for the kth parameter of γ in the rth dataset. Similarly, we calculate the average absolute relative bias (AARBias):

(6) $$\begin{array}{ll} AARBias(\gamma ) & =\frac{\sum_{k=1}^{d}\left|\sum_{r=1}^{500}{\hat{\gamma }}_{k}^{\left(r\right)}/500-\gamma_{k}\right|}{d\sum_{k=1}^{d}|\gamma_{k}|}, \end{array}$$

and the average relative standard deviation (ARSD):

(7) $$\begin{array}{ll} ARSD(\gamma ) & =\frac{\sqrt{\sum_{r=1}^{500}\sum_{k=1}^{d}{\left({\hat{\gamma }}_{k}^{\left(r\right)}-{\bar{\gamma }}_{k}\right)}^{2}/500}}{d\sum_{k=1}^{d}|\gamma_{k}|}, \end{array}$$

where ${\bar{\gamma }}_{k}=\sum_{r=1}^{500}{\hat{\gamma }}_{k}^{\left(r\right)}/500$. Finally, to investigate how the estimation of coefficients behaves after taking into account the participant‐level information, we calculate the participant‐specific root mean squared error (PSRMSE):

(8) $$\begin{array}{l} PSRMSE=\sum_{r=1}^{500}||\left(t\alpha +X^{em}\gamma \right)-\left(t{\hat{\alpha }}^{\left(r\right)}+X^{em}{\hat{\gamma }}^{\left(r\right)}\right)||/500\sqrt{N}, \end{array}$$

where $\hat{\alpha }$ is the posterior mean of α, $\hat{\gamma }$ is the posterior mean of γ, “||·||” is the Euclidean norm, and N is the number of participants across trials. The participant‐specific root mean squared error for effect moderation (PSRMSE(EM)) is defined as:

(9) $$\begin{array}{l} PSRMSE(EM)=\sum_{r=1}^{500}||X^{em}\gamma -X^{em}{\hat{\gamma }}^{\left(r\right)}||/500\sqrt{N}. \end{array}$$

ARRMSE, AARBias, and ARSD are scalar metrics free from unit and dimension to assess the estimations of treatment effect moderation, with smaller values indicating better performance. In contrast, PSRMSE and PSRMSE(EM) are not free from unit and dimension, but reflect the estimation risk at a participant‐level by taking into account the participant's information via $x_{ij}$. We expect that shrinkage methods will yield smaller values of the risk metrics, including ARRMSE, PSRMSE, and PSRMSE(EM), particularly in challenging cases such as high between‐trial variability or high model sparsity. In addition, we expect that the CMG methods result in better performance than other methods.

### 5.3 Simulation Results

**FIGURE 1:**
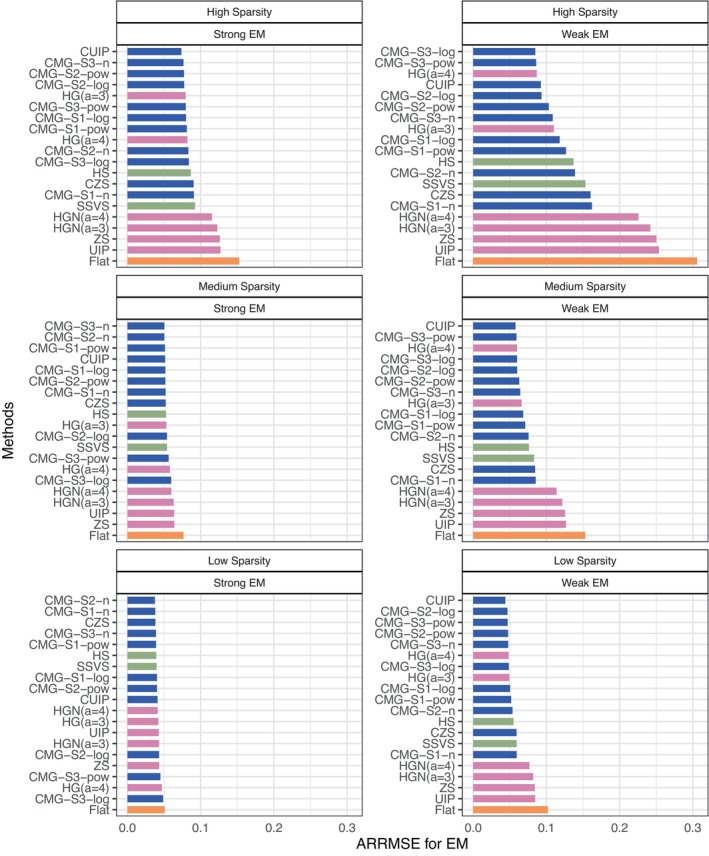
ARRMSE of moderation effects (γ) across 20 methods under a combination of three model sparsity settings (from top to bottom panels) and two magnitudes of effect moderation settings (left and right panels) with high between‐trial variability and correlated covariates. In each panel, methods are ordered by their performance. The blue, pink, green, and orange bars correspond to the CMG, NMG, HS, and SSVS, and Flat methods, respectively.

Figure 1 shows how ARRMSE varies across methods under a combination of three model sparsity settings (from top to bottom panels) and two magnitudes of effect moderation settings (left and right panels) with high between‐trial variability and correlated baseline covariates. Overall, the CUIP, CMG‐S3‐n, CMG‐S2‐pow, CMG‐S1‐log, and CMG‐S1‐pow methods are ranked among the top 10 methods across all six scenarios with small ARRMSE, indicating that these methods are less sensitive to misspecification of the true magnitude of the moderator effect and model sparsity. In contrast, the HGN (a=3), HGN (a=4), ZS, UIP and Flat methods are ranked in the bottom 10 with large ARRMSE, and the Flat method uniformly performs the worst across all cases. This highlights the need for calibration of NMG methods. Furthermore, as model sparsity increases (from top to bottom panels) or as the magnitude of the moderator decreases (from left to right panels), the CMG methods tend to produce smaller ARRMSE values, and the differences among the methods become more pronounced. Specifically, the CMG methods perform better than other methods with respect to ARRMSE under the setting of high model sparsity and small moderation effect (see bottom right panel). In this setting, among the CMG methods (results shown with blue bars in each figure), the CMG‐S3‐log method provides the smallest ARRMSE, corresponding to the maximum level of prior shrinkage and closely reflecting the true data generation mechanism.

**FIGURE 2:**
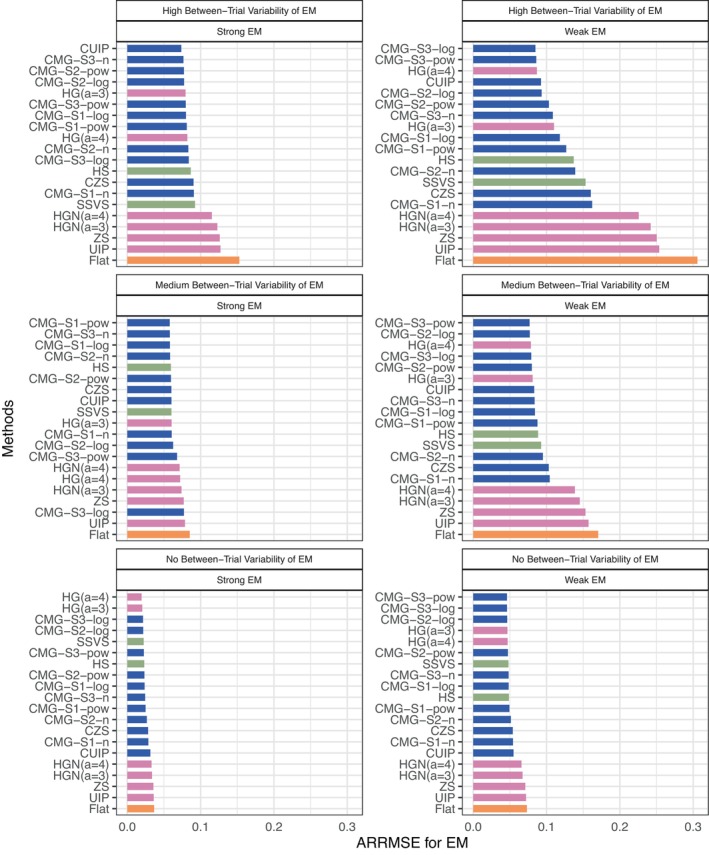
ARRMSE of moderation effects (γ) across 20 methods under the combination of three between‐trial variability settings (from top to bottom panels) and two magnitudes of effect moderation settings (left and right panels) with high model sparsity and correlated covariates. In each panel, methods are ordered by their performance. The blue, pink, green, and orange bars correspond to the CMG, NMG, HS, and SSVS, and Flat methods, respectively.

Figure 2 presents how ARRMSE changes across methods under a combination of three between‐trial variability settings (from top to bottom panels) and two magnitudes of effect moderation settings (left and right panels) with high model sparsity and correlated covariates. Overall, the CMG‐S3‐n, CMG‐S2‐pow, HG (a=3), and CMG‐S1‐log methods are consistently ranked within the top 10 with small ARRMSE. In contrast, the CMG‐S1‐n, HGN (a=3), HGN (a=4), ZS, UIP, and Flat methods are ranked within the bottom 10 in terms of larger ARRMSE. As expected, the Flat method is the worst across these cases. Moreover, as the between‐trial variability decreases (from top to bottom panels) or the magnitude of the moderation effect increases (from right to left), the distinctions among methods and the advantages of CMG methods become less evident. Specifically, the setting of high between‐trial variability and weak effect moderation (see top right panel) presents the most substantial differences among methods. In this setting, the best CMG prior method, CMG‐S3‐log, reduces ARRMSE by 72.35%, 38.25%, and 44.71% compared to the Flat, HS, and SSVS methods, respectively, further consolidating the impacts of the magnitude of the moderation effect in Figure 1.

**FIGURE 3:**
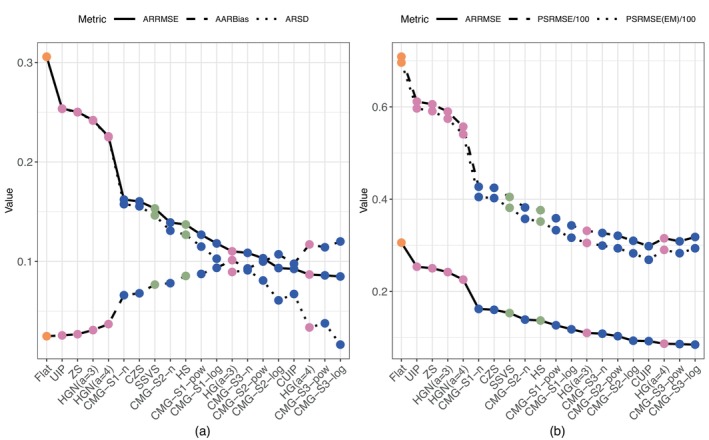
Simulation results under the setting of high model sparsity, high between‐trial variability, weak effect moderator, and correlated covariates. Panel (a) shows ARRMSE (solid), ARSD (dotted), and AARBias (dashed) of moderation effects (γ) across 20 methods with a descending order of ARRMSE from left to right. Panel (b) shows ARRMSE (solid) of γ, PSRMSE/100 (dashed) of α and γ, and PSRMSE(EM)/100 (dotted) of γ across 20 methods with a descending order of ARRMSE from left to right. The blue, pink, green, and orange dots correspond to the CMG, NMG, HS, and SSVS, and Flat methods, respectively.

Figure 3 compares the dynamic trends of multiple metrics across the 20 methods under the setting of the high model sparsity, weak effect moderators, correlated baseline covariates, and high between‐trial variability of effect moderation. Panel (a) overlays ARRMSE, AARBias, and ARSD, and presents methods in the descending order of ARRMSE from left to right. As ARRMSE decreases, AARBias tends to increase while ARSD tends to decrease. The trend of ARRMSE is primarily driven by ARSD since the decrease in ARSD is dominant over the increase in AARBias, indicating the bias‐variance trade‐off. For example, comparing the CMG‐S3‐log method (far right) with the Flat method (far left), ARRMSE reduces more than 0.2 units at the cost of less than a 0.1 unit increase in AARBias. In addition, ARRMSE drops sharply when moving sequentially from the Flat method to CMG‐S1‐n method. All NMG methods (pink dots) on the left side of the CMG‐S1‐n prior improve ARRMSE at a negligible sacrifice in bias, since the sacrifice is so small that we can not visually distinguish ARSD and ARRMSE in the figure. In contrast, the ARRMSE decreases more slowly when shifting from the CMG‐S1‐n to CMG‐S3‐log method, accompanied by a noticeable rise in AARBias. However, the faster decline in ARSD offsets the AARBias increase, resulting in an overall reduction in ARRMSE.

Panel (b) in Figure 3 further compares the parameter‐level estimation (measured by ARRMSE), and participant‐level estimation (measured by PSRMSE and PSRMSE(EM)), and presents methods in the descending order of ARRMSE from left to right. To visualize the trends together on a reasonable scale, we plot PSRMSE/100 and PSRMSE(EM)/100 alongside ARRMSE. Overall, the relative performance of methods in the participant‐level estimation aligns with their parameter‐level performance. However, PSRMSE and PSRMSE(EM) exhibit sharper reductions compared to ARRMSE, highlighting the greater benefits of using the CMG methods for participant‐level estimation. This also illustrates how a small improvement in parameter‐level risk can translate into a larger impact in participant‐level metrics. Notably, the differences between PSRMSE and PSRMSE(EM) are minimal for the Flat, UIP, ZS, and HGN methods, but become more pronounced for the other methods. This widening difference reflects more effective shrinkage in the moderation effects, accounting for participant‐level characteristics.

Finally, as a complement to the scenarios shown in Figure 1, we provide additional performance metrics, including AARBias (Figure S5) and ARSD (Figure S6), as well as ARRMSE for uncorrelated covariates as a counterpart to Figure 2 (Figure S7). Figures S4–S7 are available in Supporting Information Section S2. Further simulation scenarios (results not shown) demonstrate patterns similar to those observed in Figures 1, 2, 3: the proposed methods show greater advantages in terms of ARRMSE as the correlations among covariates become stronger, between‐trial variability and sparsity become higher, and the magnitude of moderation effects becomes smaller. Overall, increased noise in the data necessitated stronger shrinkage, thus favoring methods capable of greater shrinkage. Additional detailed results are available from the authors upon request.

## 6 Data Analysis

### 6.1 Major Depressive Disorder Dataset

#### 6.1.1 Data Description

We demonstrate our methods using a real IPD‐MA data example of four randomized controlled trials (RCTs) comparing efficacy between duloxetine and vortioxetine for patients having major depressive disorder (MDD) [43, 44, 45, 46]. Detailed description of these trials can be found elsewhere [47].

The primary outcome is the change in Montgomery‐Åsberg Depression Rating Scale (MADRS) total score from baseline to the last observed follow‐up. A higher MADRS score indicates a more severe symptom. Twelve baseline covariates are considered as potential effect moderators: age, sex (female or male), smoking status (ever smoked or never smoked), weight, baseline MADRS score, baseline Hamilton Anxiety Rating (HAM‐A) score, three comorbidity indicators for diabetes mellitus (DM), hypothyroidism, and anxiety, and three medication indicators for antidepressant, antipsychotic, and thyroid medication. For simplicity, we excluded 3 participants having incomplete baseline covariates, resulting in a total of 1844 participants.

#### 6.1.2 Model Fitting and Comparison Details

This data analysis aims to assess meaningful effect moderators that alter treatment effects between duloxetine and vortioxetine. We fit the IPD‐MA models in (2) with and without between‐trial variability for moderation effects, using 20 methods listed in Table 1. To evaluate whether the inclusion of between‐trial variability improves the model (i.e., with and without $u_{i}$), we use the deviance information criterion (DIC) [48], with smaller DIC values indicating better model fit.

The IPD‐MA models without between‐trial variability of moderation effects fit our data slightly better than those with between‐trial variability, as indicated by smaller DIC values across all methods. Given the typically small number of moderators in practice, in addition to the commonly used Flat method, we select the HG (a=4) and CMG‐S3‐pow methods based on the best approaches identified through comparable simulation settings, as shown in the two lower panels of Figure 2. Results for the existing HS and SSVS methods are also provided in Supporting Information Section S3 (Figure S8, Tables S1, and S2).

We then utilize the three selected methods to illustrate the application of the proposed methods in assessing effect moderation. Specifically, we focus on the posterior standard deviation (SD), 95% credible intervals (CI), and the marginal posterior density plots of the covariate‐by‐treatment interaction term $\gamma_{k}$ in (2). We check whether the 95% CI excludes 0 to identify important effect moderators. However, the 95% CI is often overly wide, potentially excluding meaningful effect moderators. This may occur when the sample size is not large enough or when there is high variability in the data, which is very common in multiple studies. As a complementary measure, we calculate posterior probabilities of $\gamma_{k}$ that falls within the interval $\left(-\sqrt{VAR(\gamma_{k}|y)},\sqrt{VAR(\gamma_{k}|y)}\right)$, and denote this quantity as $p_{\gamma_{k}}$, where $p_{\gamma_{k}}=P\left(|\gamma_{k}|<\sqrt{VAR(\gamma_{k}|y)}|y\right)$. A larger posterior probability of being in this interval indicates a weaker or less important effect moderator. Furthermore, the identification of important effect moderators based on such posterior probability with a threshold is known as the scaled neighborhood criterion [49]. Note that this measure is included to complement the estimated moderation effects, and the goal of this work is not to select variables.

Accordingly, for each method, the scaled neighborhood criterion may identify a different set of important moderators with conclusions varying based on the chosen cutoff. In the absence of a specific guideline or prior information, we adopt a threshold of 0.5, following the recommendation of [49]. Specifically, if $p_{\gamma_{k}}<0.5$, we consider $\gamma_{k}$ as an important moderation effect. This implies that there is a less than 50% chance that the moderator lies near zero conditional on this dataset, and thus indicates the moderator is more likely to have a meaningful impact. Compared to the 95% CI, the scaled neighborhood criterion typically yields narrower intervals. Simulations by [49] also show that, while 95% CI tends to deliver higher sensitivity, the scaled neighborhood criterion is more likely to outperform in specificity, under comparable conditions.

### 6.2 Data Analysis Results

Table 2 displays the posterior means, SDs and 95% CIs for key model coefficients, including the conditional treatment effect (α), moderation effects (covariate‐by‐treatment interaction γ), and between‐trial variability of α ($\tau_{\alpha }^{2}$), estimated using the Flat, HG (a=4), and CMG‐S3‐pow methods. The conditional treatment effect estimates are consistent across these methods, with 95% CIs excluding 0, and the HG (a=4) and CMG‐S3‐pow methods producing slightly narrower CIs. For the moderation effects, the three methods agree on the directions for most moderators, except for hypothyroidism ($\gamma_{8}$) and antipsychotic ($\gamma_{11}$). While all moderation effect estimates have 95% CIs that include 0 across the three methods, both HG (a=4) and CMG‐S3‐pow methods greatly improve their precisions, reducing posterior SDs by about 50%. The HG(a=4) and CMG‐S3‐pow methods perform similarly, where HG(a=4) shows slightly smaller SDs, but CMG‐S3‐pow is favored by smaller DIC.

**TABLE 2:** Posterior means (posterior SDs) and 95% CIs of selected model coefficients (α, γ and $\tau_{\alpha }^{2}$) estimated using the Flat, HG (a=4), and CMG‐S3‐pow methods from the IPD‐MA real data analysis.

Selected coefficients	Flat	HG (a=4)	CMG‐S3‐pow
α **: Conditional treatment effect**	2.64 (1.08)	2.55 (0.85)	2.56 (0.93)
	(0.44, 4.63)	(0.93, 4.20)	(0.82, 4.29)
$\gamma_{1}$ **: Sex‐by‐treatment**	−0.04 (1.08)	−0.08 (0.49)	−0.08 (0.53)
	(−2.12, 2.05)	(−1.19, 0.91)	(−1.35, 1.02)
$\gamma_{2}$ **: Age‐by‐treatment**	0.07 (0.04)	0.02 (0.03)	0.03 (0.03)
	(−0.003, 0.15)	(−0.01, 0.09)	(−0.02, 0.1)
$\gamma_{3}$ **: Smoking status‐by‐treatment**	−1.28 (1.07)	−0.34 (0.63)	−0.39 (0.67)
	(−3.42, 0.78)	(−1.97, 0.61)	(−1.93, 0.66)
$\gamma_{4}$ **: Weight‐by‐treatment**	−0.01 (0.02)	−0.003 (0.01)	−0.003 (0.01)
	(−0.06, 0.04)	(−0.03, 0.02)	(−0.03, 0.02)
$\gamma_{5}$ **: Baseline MADRS score‐by‐treatment**	0.11 (0.13)	0.03 (0.07)	0.04 (0.07)
	(−0.14, 0.37)	(−0.08, 0.20)	(−0.09, 0.21)
$\gamma_{6}$ **: Baseline HAM‐A score‐by‐treatment**	0.04 (0.09)	0.02 (0.05)	0.02 (0.05)
	(−0.14, 0.22)	(−0.06, 0.13)	(−0.06, 0.14)
$\gamma_{7}$ **: DM‐by‐treatment**	−3.78 (3.01)	−0.95 (1.78)	−1.24 (2.03)
	(−9.56, 2.36)	(−5.26, 1.96)	(−6.07, 2.09)
$\gamma_{8}$ **: Hypothyroidism‐by‐treatment**	−0.45 (3.92)	0.03 (1.27)	−0.002 (1.48)
	(−8.14, 7.36)	(−2.47, 2.77)	(−3.12, 3.2)
$\gamma_{9}$ **: Anxiety‐by‐treatment**	3.54 (2.88)	1.15 (1.86)	1.25 (1.97)
	(−2.17, 9.19)	(−1.93, 5.60)	(−1.86, 5.91)
$\gamma_{10}$ **: Antidepressant‐by‐treatment**	2.04 (1.33)	0.59 (0.83)	0.69 (0.89)
	(−0.61, 4.7)	(−0.58, 2.64)	(−0.61, 2.76)
$\gamma_{11}$ **: Antipsychotic‐by‐treatment**	−0.73 (1.72)	0.10 (0.80)	0.08 (0.9)
	(−4.11, 2.61)	(−1.52, 1.94)	(−1.95, 2.06)
$\gamma_{12}$ **: Thyroid medication‐by‐treatment**	0.51 (4.85)	0.05 (1.62)	0.06 (1.77)
	(−8.91, 9.76)	(−3.46, 3.41)	(−3.74, 4.05)
$\tau_{\alpha }^{2}$ **: Between‐trial variability of treatment**	1.34 (1.01)	1.06 (0.83)	1.09 (0.89)
	(0.07, 3.86)	(0.05, 3.10)	(0.07, 3.32)

**FIGURE 4:**
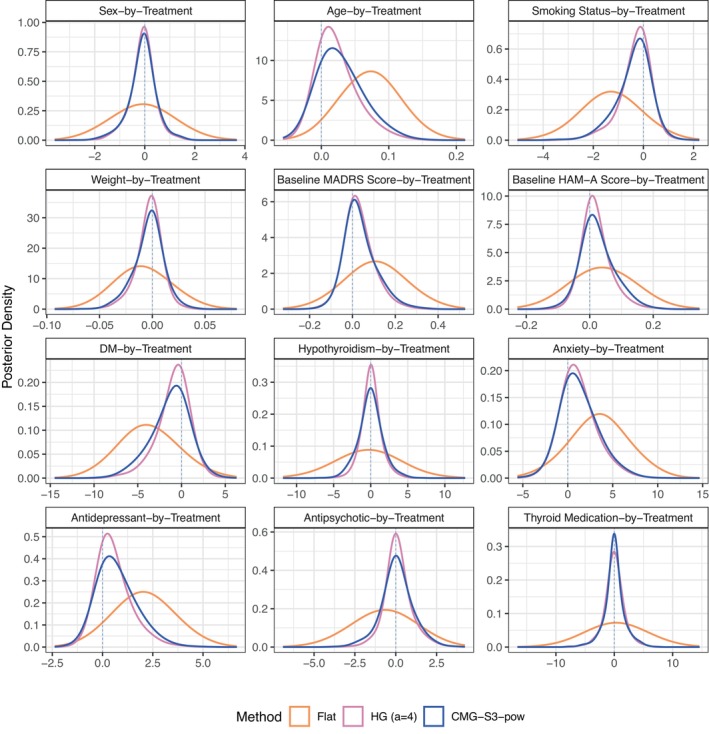
Posterior density plots for moderation effects, covariate‐by‐treatment interactions ($\gamma_{1},\cdots ,\gamma_{12}$), under the HG (a=4), CMG‐S3‐pow, and Flat methods from the IPD‐MA real data analysis.

Figure 4 shows the marginal posterior distributions of $\gamma_{k}$ under the Flat (orange lines), HG (a=4) (pink lines), and CMG‐S3‐pow (blue lines) methods. We assess the importance of each effect moderator by visually comparing the concentration and deviation from the null across methods. If the posterior density of $\gamma_{k}$ remains shifted from zero under strong shrinkage (i.e., CMG‐S3‐pow or HG (a=4) methods), it suggests that the evidence from the data is strong enough to overcome the shrinkage, and thus indicates a potentially important moderator. This is reinforced if $\gamma_{k}$ from the Flat method is concentrated at a nonnull value, indicating an optimistic and important signal. Conversely, if $\gamma_{k}$ is centered around zero in both the strong shrinkage and Flat methods, as the posterior aligns with the prior's expectation of no effect. For example, for the coefficient of sex‐by‐treatment, the posterior densities of three methods are perfectly concentrated around zero, indicating that sex is not an important effect moderator. In contrast, for the coefficient of age‐by‐treatment, its posterior densities vary greatly across methods, with the Flat method showing the widest distribution and peaking above the null. The HG (a=4) and CMG‐S3‐pow methods produce narrower posterior distributions, concentrated at a smaller value compared to that under the Flat method. This suggests evidence of moderation effect even under strong regularization, indicating that age is a relatively important moderator. Similarly, smoking status, DM, anxiety, and antidepressants are likely to be important moderators. It is straightforward that sex, hypothyroidism, and thyroid medication are not considered as important moderators, as all three methods are symmetrically centered around the null. Finally, while the Flat method shows concentrations for weight, baseline MADRS score, baseline HAM‐A score, and antipsychotic that are away from zero, both the HG (a=4) and CMG‐S3‐pow methods are strongly concentrated around zero, suggesting little evidence for the importance of these moderators.

Finally, Table 3 presents posterior probabilities of coefficients $p_{\gamma_{k}}$ for all covariate‐by‐treatment interactions under the Flat, HG(a=4), and CMG‐S3‐pow methods. To identify important moderators, we compare the posterior probability with a threshold such as 0.5. For example, for the sex‐by‐treatment interaction ($\gamma_{1}$), the Flat ($p_{\gamma_{1}}=0.68$), HG (a=4) ($p_{\gamma_{1}}=0.76$), and CMG‐S3‐pow methods do not identify sex as an important effect moderator, since the corresponding posterior probabilities are greater than 0.5. Similarly, the Flat method identifies Age, DM, Anxiety, and Antidepressant as important moderators, while HG (a=4) and CMG‐S3‐pow do not identify any important moderators. This implies the strong shrinkage effects of the HG (a=4) and CMG‐S3‐pow methods, where HG (a=4) method generally shows higher posterior probabilities compared to the CMG‐S3‐pow method. To assess moderators under various shrinkage levels, across 20 methods, 14 methods identify age as an important effect moderator, 8 methods identify antidepressant use, and 6 methods identify smoker, DM, and anxiety as important moderators. The remaining covariate‐by‐treatment interactions are not considered by any method. These results align with the conclusions drawn in Table 2 and Figure 4.

**TABLE 3:** The scaled neighborhood criterion based posterior probabilities $p_{\gamma_{k}}$ for covariate‐by‐treatment interaction ($\gamma_{1},\ldots ,\gamma_{12}$) from the IPD‐MA real data analysis.

Covariate‐by‐treatment coefficients	Flat	HG (a=4)	CMG‐S3‐pow
$\gamma_{1}$ **: Sex‐by‐treatment**	0.68	0.76	0.75
$\gamma_{2}$ **: Age‐by‐treatment**	0.20	0.63	0.58
$\gamma_{3}$ **: Smoking status‐by‐treatment**	0.58	0.74	0.70
$\gamma_{4}$ **: Weight‐by‐treatment**	0.66	0.74	0.73
$\gamma_{5}$ **: Baseline MADRS score‐by‐treatment**	0.53	0.73	0.71
$\gamma_{6}$ **: Baseline HAM‐A score‐by‐treatment**	0.64	0.74	0.72
$\gamma_{7}$ **: DM‐by‐treatment**	0.38	0.71	0.69
$\gamma_{8}$ **: Hypothyroidism‐by‐treatment**	0.68	0.76	0.74
$\gamma_{9}$ **: Anxiety‐by‐treatment**	0.40	0.68	0.67
$\gamma_{10}$ **: Antidepressant‐by‐treatment**	0.30	0.69	0.64
$\gamma_{11}$ **: Antipsychotic‐by‐treatment**	0.65	0.77	0.74
$\gamma_{12}$ **: Thyroid medication‐by‐treatment**	0.68	0.76	0.77

## 7 Discussion

In this work, we have proposed the CMG methods in IPD‐MA to assess treatment effect moderation. The proposed methods provide a novel series of shrinkage priors that are tailored for multiple studies. Both NMG and CMG incorporate study‐level information and a moderator‐level shrinkage parameter. CMG additionally refines this framework by leveraging more detailed study‐level information through a study‐specific sample size tuning function and offering greater flexibility in shrinkage levels. Our simulation study compares CMG and NMG to two existing shrinkage methods, focusing on the estimation performances of these priors, which have been rarely explored in the mixtures of g‐priors. The simulation study indicates that CMG methods outperform other methods in challenging settings, including high between‐study variability, high model sparsity, weak moderation effects, and correlated design matrices in terms of ARRMSE. We further demonstrate the application of the proposed methods to assess moderation effects of two active treatments for major depressive disorder.

The CMG methods in (4) can be extended by replacing $g_{k}$ with $g_{k,i}$ to leverage study‐level contribution at a more concrete level.

(10) $$\begin{array}{ll} \gamma_{k} & |\Lambda^{⋆},t,x_{\left[k\right]}∼N\left(0,{\left[{\left(t\circ x_{\left[k\right]}\right)}^{\prime }\Lambda^{⋆}(t\circ x_{\left[k\right]})\right]}^{-1}\right),\\ & \Lambda^{⋆}=diag\left(\sigma_{1}^{-2}g_{k,1}^{-1}f{\left(n_{1}\right)}^{-1}1_{n_{1}},\cdots ,\right.\\ & \;\left.\sigma_{I}^{-2}g_{k,I}^{-1}f{\left(n_{I}\right)}^{-1}1_{n_{I}}\right),\;g_{k,i}>0. \end{array}$$

Although (10) offers more flexibility, it requires a large sample size to compensate for the increasing number of parameters. Our additional simulations (results not shown) indicate that the benefits gained using (10) are minimal, and thus we recommend CMG in (4). Moreover, the CMG methods have broad potential applications, as it is grounded in the mixtures of g‐priors, which has a substantial body of literature. For example, mixtures of g‐priors share conceptual links with ridge regression [50] and power prior [51]. Over the years, multiple variants of mixtures of g‐priors have been developed, including but not limited to extensions to analysis of variance design [52], cases where parameter dimensions outnumber sample sizes [53], generalized linear regression model [54], and many others [55, 56, 57].

Our simulation results conclude that the CMG methods perform as well as or better than other methods in terms of higher efficiency and lower risks (e.g., ARRMSE). These advantages are especially evident in cases with sparse models, weak effect moderation, high between‐study variability, and correlated design matrices. The CMG methods accommodate a broad range of shrinkage levels, offering a full assessment of the risk measures in estimating moderation effects, varying from the most conservative to the most optimistic method. This flexibility allows researchers to tailor the prior specification according to the desired level of shrinkage. For instance, if a researcher believes that more than 50% shrinkage is unacceptable, one might exclude certain methods like S3 from consideration. Our results suggest that the Flat method is uniformly worse than any type of shrinkage methods, indicating that the shrinkage methods are more suitable for assessing treatment effect moderation.

In our data analysis, it is important to note that two original trials used duloxetine as a reference medication, excluding patients with prior nonresponse. This exclusion criterion did not apply to the other two MDD trials, potentially introducing bias in the estimated conditional treatment effects of duloxetine and vortioxetine. These differences highlight the importance of incorporating study‐specific parameters when constructing priors using multiple studies (e.g., the study‐level sample size tuning functions of the CMG methods). We investigate multiple measures and methods to assess the importance of moderators. For example, while the 95% CIs did not identify any important moderators with all methods, the scaled neighborhood criterion identified several potentially important ones, with age being the most frequently selected. The Flat method, the most optimistic, identified five moderators, and a method with minimal prior shrinkage (e.g., CMG‐S1‐n) selected two. In contrast, strong shrinkage methods (e.g., CMG‐S3‐log and CMG‐S3‐pow) identified none, reflecting the most conservative conclusions. Therefore, it is important to consider the spectrum of shrinkage levels, as they reflect varying degrees of optimism or conservatism, enabling more informed decision‐making and allowing us to interpret results with an awareness of these differences rather than relying solely on a single method.

In addition, we reflect on several limitations and discuss directions for future research. First, in the CMG methods, we incorporate $f(n_{i}|p_{i})$ to account for study‐level variation, allowing the moderator‐level tuning parameter $g_{k}$ to more effectively identify moderators. For instance, a large estimated $g_{k}$ may suggest an important moderator, while a small $g_{k}$ could indicate a nonimportant one. Although $g_{k}$ performs well in distinguishing important moderators in studies with strong signals (results not shown), its effectiveness declines as study designs become more complex. Since $g_{k}$ and $f(n_{i}|p_{i})$ jointly determine the final shrinkage effect with $g_{k}$ playing a dominant role, future efforts will focus on refining the CMG methods by integrating $g_{k}$ and $f(n_{i}|p_{i})$ into a unified quantity to identify important moderators.

Second, in the data analysis, we complement the posterior density plots with the scaled neighborhood criterion to identify important moderators. However, this criterion relies only on posterior SDs to set the size of the neighborhood (i.e., $\sqrt{VAR(\gamma_{k}|y)}$), ignoring the posterior means (i.e., location). Incorporating both location and scale is needed because the distributions of important moderators are characterized not only by variability but also by the extent to which their estimated effects differ from zero. Exploring a neighborhood constructed using both posterior means and standard deviations may provide a more effective way by considering both the magnitude and uncertainty of their estimated effects.

Third, a fundamental limitation in analyzing real data is that the true underlying mechanisms or actual moderation effects are unknown. Consequently, our simulation studies do not enumerate all scenarios that may be encountered in practice. In this setting, researchers have several ways to leverage the proposed methods. Real data‐based simulations can provide additional insight into which shrinkage methods are most suitable for a given dataset [58]. The choice among the proposed methods can also be guided by the availability of domain knowledge. When such knowledge is limited or unavailable, we recommend CMG‐S3‐n or CMG‐S2‐pow, which demonstrated robust performance across scenarios (consistently ranked among the top 10 in Figures 1, 2, and S7), with CMG‐S3‐n exhibiting smaller biases. When additional domain insight into the expected magnitude of moderation effects is available, CMG‐S3‐n is more suitable for stronger effects, whereas CMG‐S2‐pow is preferable for weaker effects; if few true moderators with weak signals are anticipated, applying stronger shrinkage through CMG‐S3‐log may be advantageous. Crucially, if moderation effects are expected to be small but nonzero, we caution against relying on SSVS. As our simulations indicate, the spike and slab formulation of SSVS tends to aggressively over‐shrink weak signals into the spike component, making it less suitable for estimating these small effects. For example, across the 14 weak effect scenarios shown in Figures 1, 2, S3, and S4, SSVS is consistently among the bottom 10 methods due to its larger ARRMSE. In addition, rather than relying solely on traditional 95% credible intervals, researchers can assess moderation effects more comprehensively by comparing posterior distributions obtained under priors with different shrinkage levels to understand the most conservative and optimistic estimation. Supplementary diagnostic tools, such as prior predictive checks or predictive accuracy metrics, can further aid researchers in understanding competing approaches [59]. Finally, researchers can leverage the full spectrum of shrinkage methods by ensembling various shrinkage approaches to provide a unified estimation for moderation effects [60, 61]. However, the accuracy, robustness, and computational feasibility of such ensemble methods require further investigation, especially within the IPD‐MA context.

Finally, although the CMG methods show promising performance in estimating moderation effects at both the parameter and participant levels, an important challenge is to translate these estimates into actionable, individualized treatment recommendations. From a clinical decision‐making perspective, the key question is: for a new patient, which treatment, among those studied in the completed trials, is expected to yield the most favorable outcome? Thus, one of our future directions is to consider a predictive framework in which the baseline characteristics of the new patient are assumed to be comparable to those of participants in the completed trials, so that CMG‐based individualized treatment assessment can be used to quantify the benefit of one treatment over another. Relevant evaluation criteria include both global prediction metrics (e.g., prediction error) and operating characteristics for detecting true moderators (e.g., the true positive rate) [62, 63, 64, 65]. A comprehensive assessment will require additional targeted simulation studies to investigate the robustness and practical utility of CMG methods for guiding personalized treatment decisions.

## Funding

This paper is based on research using data from data contributors, Takeda, and Lundbeck and, that have been made available through Vivli Inc. Vivli has not contributed to or approved, and is not in any way responsible for, the contents of this publication.

The study was funded by the Patient‐Centered Outcomes Research Institute (PCORI) through PCORI Award ME‐2020C321145 and the National Institute of Mental Health (NIMH) through Award R01MH126856.

Disclaimer: Opinions and information in this content are those of the study authors and do not necessarily represent the views of PCORI or NIMH. Accordingly, PCORI and NIMH cannot make any guarantees with respect to the accuracy or reliability of the information and data. Furthermore, this paper is based on research using data from data contributors, Takeda, and Lundbeck and, that have been made available through Vivli Inc. Vivli has not contributed to or approved, and is not in any way responsible for, the contents of this publication.

## Conflicts of Interest

The authors declare no conflicts of interest.

## Supporting information




**Data S1**: Supporting Information.

## Data Availability

Real data used in the applied example portion of the article were analyzed through Vivli. Restrictions apply to the availability of these data, which were used under license for this study.
