# Supplementary material for: Bayesian Hierarchical Models With Calibrated Mixtures of g‐priors for Assessing Treatment Effect Moderation in Meta‐Analysis
Source: Stat Med. 2026 Apr 2;45(8-9):e70510. doi: 10.1002/sim.70510 (PMC13044573; doi:10.1002/sim.70510)
Supplement: Supplementary file 1 — Data S1: Supporting Information. [file SIM-45-0-s001.pdf]

Supplementary Material for "Bayesian hierarchical models with calibrated mixtures of g-priors for assessing treatment effect moderation in meta-analysis"

Supplementary Material

Qiao Wang *
Department of Public Health
East Carolina University Brody School of Medicine
wangqi25@ecu.edu

Hwanhee Hong
Department of Biostatistics and Bioinformatics
Duke University School of Medicine
hwanhee.hong@duke.edu

Section S1 Study-level Sample-Size Tuning Function in Section 4.2.2

This section provides additional rationale behind the proposed study-level sample-size tuning function presented in Section 4.2.2 of the main manuscript. We first explain the importance for incorporating $f(n_i|p_i)$ into the prior variance, and then describe the reasoning that guided the choice of its functional form, as illustrated in Figures S1-S3.

S1.1 Rationale for Incorporating $f(n_i|p_i)$ in the Prior Variance

The study-level sample-size tuning function $f(n_i|p_i)$ directly calibrates how each study contributes to the prior variance for each moderation effect γ_k, where n_i denotes the sample size and p_i is a tuning parameter for study i. Under the calibrated mixtures of g-priors defined in Equation (4) of the main manuscript, the prior variance for kth moderation effect can be rewritten as:

$$Var(\gamma_k|g_k, \boldsymbol{\Lambda}^{\star}, p_i, \boldsymbol{t}, \boldsymbol{x}_{[k]}) = g_k/S_f, \text{ and } S_f = \sum_{i=1}^{I} \frac{n_i}{\sigma_i^2 f(n_i|p_i)} \sum_{j=1}^{n_i} \frac{(t_{ij}x_{ijk})^2}{n_i}, \tag{1}$$

where σ_i is the noise parameter, t_{ij} in $\boldsymbol{t}$ is a binary treatment indicator, and x_{ijk} in $\boldsymbol{x}_{[k]}$ is the covariate for the kth moderation effect. Then, each study's contribution to the prior precision can be defined as follows:

$$s_i = \frac{n_i}{\sigma_i^2 f(n_i|p_i)} \sum_{j=1}^{n_i} \frac{(t_{ij}x_{ijk})^2}{n_i} \text{ and } \sum_{i=1}^{I} s_i = S_f. \tag{2}$$

*Corresponding Author

In equation (2), under within-study randomization ($t_{ij} \perp x_{ijk}$) with $t_{ij} \sim Bernoulli(\pi_i)$, and $\nu_{k,i} = \mathbb{E}[x_{ijk}^2] < \infty$, the law of large numbers yields:

$$\sum_{j=1}^{n_i} \frac{(t_{ij}x_{ijk})^2}{n_i} \xrightarrow{p} \mathbb{E}(tx^2) = \mathbb{E}(t)\mathbb{E}(x^2) = \pi_i \, \nu_{k,i}. \tag{3}$$

Thus, each study's contribution depends on its study design (e.g., the distribution of treatment assignment t_{ij} and baseline characteristics x_{ijk}), its noise parameter (σ_i), and the ratio of $n_i/f(n_i|p_i)$, indicating the importance of $f(n_i|p_i)$ in setting the prior variance.

S1.2 Considerations for the Functional Form of $f(n_i|p_i)$

It is not appropriate to choose $f(n_i|p_i) = 1$. $f(n_i|p_i) = 1$ implies that no sample size is leveraged in $f(n_i|p_i)$. From equations (2) and (3), the ith study's contribution s_i increases without bound as the sample size n_i grows. Consequently, the prior variance becomes excessively concentrated around zero, causing overly aggressive shrinkage for larger studies regardless of the true moderation effects. Figure S1 illustrates this phenomenon by showing the corresponding prior densities.

In Figure S1, it is evident that the choice of the sample-size tuning functions substantially affects the prior densities. The No Sample Size (NSS) ($f(n_i) = 1$) produces narrowest and most concentrated prior densities among five functions. In contrast, the study-level tuning functions (n, pow and log) yield priors that better regulate the influence of increasing sample size. This adaptive behavior can also be demonstrated with our simulation results presented in the manuscript. For example, in Figure 1 of the main manuscript, all six of the best-performing cases involve a sample-size tuning function, whereas in Figure 2, five out of six do.

It is not appropriate to choose $f(n_i|p_i) = N = \sum_{i=1}^{I} n_i$. As the total sample size for all studies N is typically large, the prior precision term S_f becomes extremely small. This leads to an excessively large prior variance, resulting in a diffuse prior distribution. Consequently, the resulting shrinkage behavior is expected to be close to that of the Flat prior, providing little shrinkage toward zero. In Figure S1, the No Tuning function (NT, $f(n_i) = N$) remains nearly flat across three sample size settings, reflecting insufficient shrinkage. In this case, even a simple tuning such as $f(n_i \mid p_i) = \sqrt{N}$ is beneficial. For instance, the CUIP achieves smaller ARRMSE values than the UIP in Figures 1 and 2 of the main manuscript. Introducing a study-level sample-size tuning function further enhances this improvement. For example, the CZS with $f(n_i \mid p_i) = n_i p_i$ performs uniformly better than the ZS in Figures 1 and 2, yielding smaller ARRMSE values and demonstrating that even a simple study-level tuning can meaningfully improve the estimation of moderation effects.

The three investigated functional forms of $f(n_i \mid p_i)$ exhibit varying sublinear growth rates. Figure S1 presents the density plots for a single moderator under the three functional forms, with the log function producing the most concentration around zero, the pow function showing intermediate concentration, and the n function yielding an almost flat distribution.

Figure S2 compares three functional forms of $f(n_i \mid p_i)$ with respect to the linear function ($n/f(n \mid p)$) as sample size increases across three choices of p. Across all three values of p, the ratio $n/f(n \mid p)$ increases sublinearly with sample size under the pow and log functions, remains constant under the linear n function, and shows the steepest rise for the log form

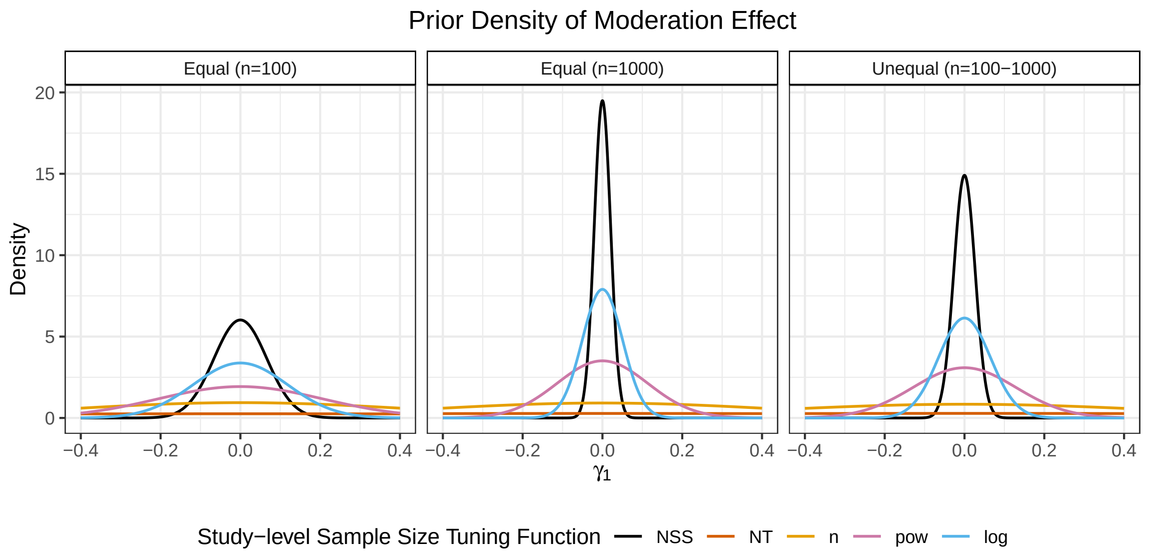

Figure S1: Demonstration of how five study-level sample-size tuning functions $f(n_i \mid p_i)$ influence prior densities of a single moderator effect (γ_1). Simulations comprise five studies with covariates $x_{ij1} \sim N(0,1)$ and treatment indicators $t_{ij} \sim$ Bernoulli(0.5). Three sample size scenarios are compared: (1) equal moderate-sized studies ($n_i = 100$), (2) equal large-sized studies ($n_i = 1000$), and (3) unequal-sized studies ($n_i = \{100, 325, 550, 775, 1000\}$). Priors are specified with $g_1 = 1$, $\sigma_i^2 = 1$, and $p_i = 0.5$. The tuning functions compared are No Sample Size (NSS, $f(n_i) = 1$, black), No Tuning (NT, $f(n_i) = \sum_{i=1}^{5} n_i = N$, red-orange), n ($f(n_i) = n_i p_i$, orange), pow ($f(n_i) = n_i^{p_i}$, reddish purple), and log ($f(n_i) = \log(n_i p_i)$, sky blue). "NSS" and "NT" are not recommended. "n", "pow" and "log" are the proposed function.

and the slowest rise for the pow form. As p increases, the growth of this ratio becomes more tempered, although the overall patterns remain relatively similar across the three functional forms.

Figure S3 investigates how the functional forms of $f(n_i \mid p_i)$ and the tuning parameter p_i jointly influence the study-level proportional contributions to prior precision across combinations of two sample size settings (equal vs. unequal) and two tuning parameter settings (equal vs. unequal) in six studies. The study-level proportional contribution to the prior precision is defined as s_i/S_f based on equations (1) and (2). Across the four panels, the contributions of individual studies under the three functional forms vary according to study size and tuning parameters, with the strongest differences appearing when sample sizes and tuning values are unequal. Overall, the functional forms exhibit greater sensitivity to tuning parameters than to sample sizes.

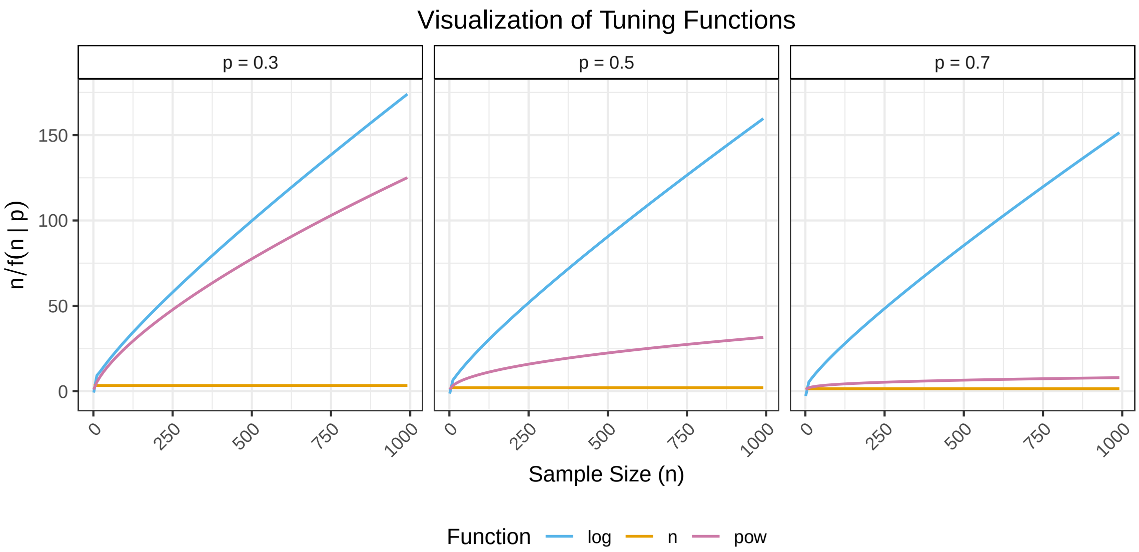

Figure S2. Comparison of three study-level sample-size tuning functions in relation to linear function (i.e., $n/f(n \mid p)$). The functions compared are n ($f(n|p) = np$, orange), *pow* ($f(n|p) = n^p$, reddish purple), and *log* ($f(n|p) = \log(np)$, sky blue). The ratio $n/f(n \mid p)$ is plotted against varying study sample sizes n (from 1 to 1000) under three tuning parameter values ($p = 0.3, 0.5, 0.7$).

Section S2 Figures for Complementary Scenarios and Metrics

This section presents additional simulation scenarios and performance metrics to complement the main manuscript including Figures S4-S7.

Figure S4 presents ARRMSE of moderation effects (γ) across 20 methods under a combination of three study size settings (from top to bottom panels) and two settings for number of studies (left and right panels) with high model sparsity, high between-trial variability, weak moderation effects and correlated covariates. In each panel, methods are arranged in increasing order of ARRMSE from top to bottom. Notably, variations in study size and the number of studies do not substantially alter the relative performance of methods in estimating moderation effects previously indicated in the main manuscript (Figure 1, bottom-right panel). Nevertheless, calibrated mixtures of g-priors with less shrinkage, such as CUIP, generally perform better with larger study sizes and greater numbers of studies. Conversely, calibrated mixtures of g-priors with greater shrinkage show better performance in scenarios involving smaller study sizes and fewer studies.

Figure S5 compares AARBias of moderation effects (γ) across 20 methods under a combination of three model sparsity settings and two magnitude of effect moderation settings with high between-trial variability and correlated covariates. This figure shows the improved efficiency of the CMG methods, and validates findings from Figures 1 and 3(a).

Figure S6 shows ARSD of moderation effects (γ) across 20 methods under a combination of three model sparsity settings and two magnitude of effect moderation settings with high between-trial variability and correlated covariates. This figure shows the sacrifice in bias using the CMG methods, but the gain in ARRMSE compensate this sacrifice, echoing findings in Figure 3(a).

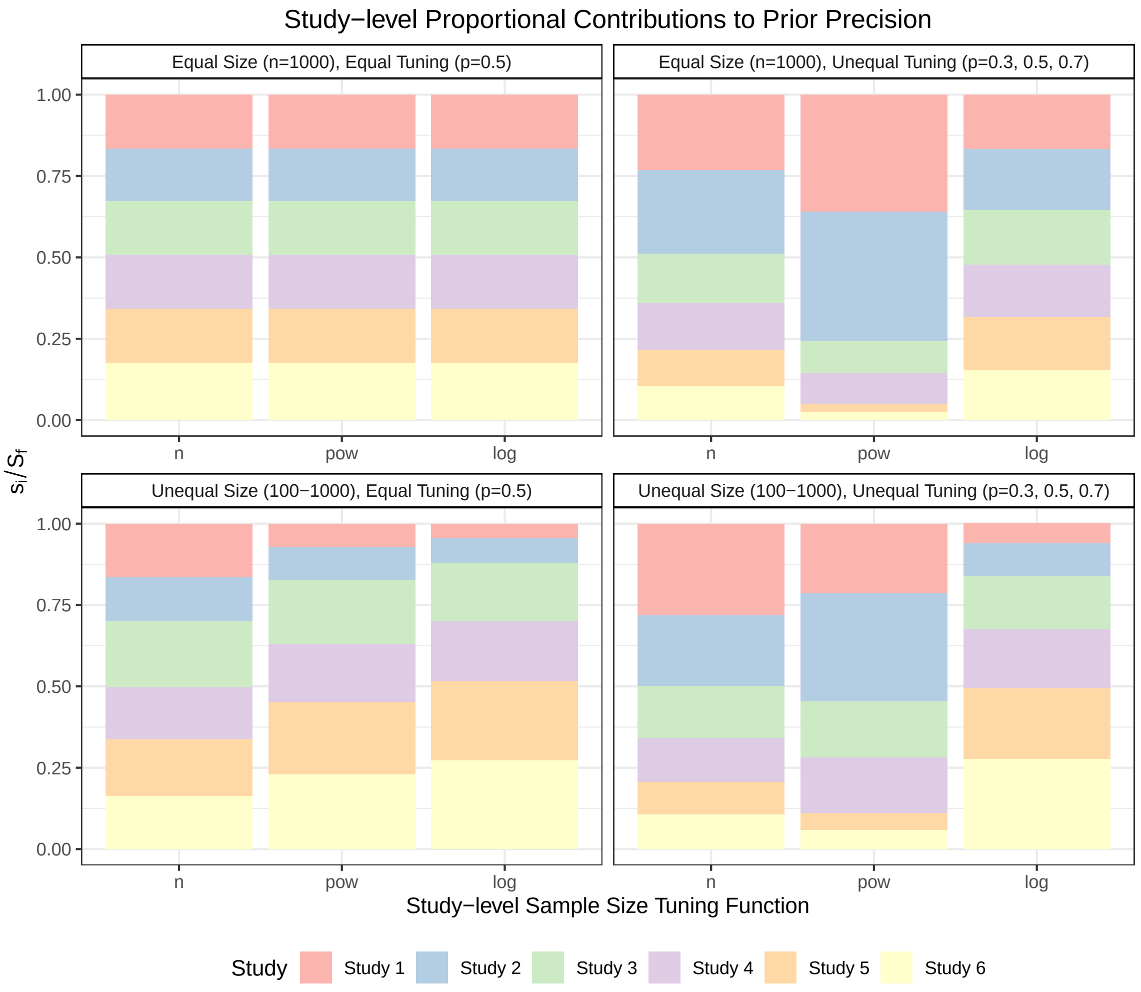

Figure S3: Demonstration of study-level proportional contributions to prior precision (s_i/S_f) under the combination of two sample size settings (equal vs. unequal) and two tuning parameter settings (equal vs. unequal) considering a single moderator. Simulations comprise six studies (Study 1: red, Study 2: blue, Study 3: green, Study 4: purple, Study 5: orange, and Study 6: yellow) with covariates $x_{ij1} \sim N(0,1)$ and treatment indicators $t_{ij} \sim \text{Bernoulli}(0.5)$. The scenarios include: (1) equal sample sizes ($n_i = 1000$) with equal tuning ($p_i = 0.5$); (2) equal sample sizes with varying tuning ($p_i = \{0.3, 0.3, 0.5, 0.5, 0.7, 0.7\}$); (3) unequal sample sizes ($n_i = \{100, 280, 460, 640, 820, 1000\}$) with equal tuning ($p_i = 0.5$); and (4) unequal sample sizes with varying tuning ($p_i = \{0.3, 0.3, 0.5, 0.5, 0.7, 0.7\}$). Three proposed tuning functions are compared: n ($f(n_i|p_i) = n_i p_i$), *pow* ($f(n_i|p_i) = n_i^{p_i}$), and *log* ($f(n_i|p_i) = \log(n_i p_i)$).

Since the main manuscript focuses primarily on correlated covariates, we provide additional ARRMSE results (Figure S7) for uncorrelated covariates as a counterpart to Figure 2, given that varying between-trial variability is an important consideration in meta-analysis. Figure S7 compares moderation effects (γ) across 20 methods, under combinations of three between-trial variability settings and two magnitudes of effect moderation, with high between-trial variability and uncorrelated covariates. The overall trends remain consistent with those observed for correlated covariates.

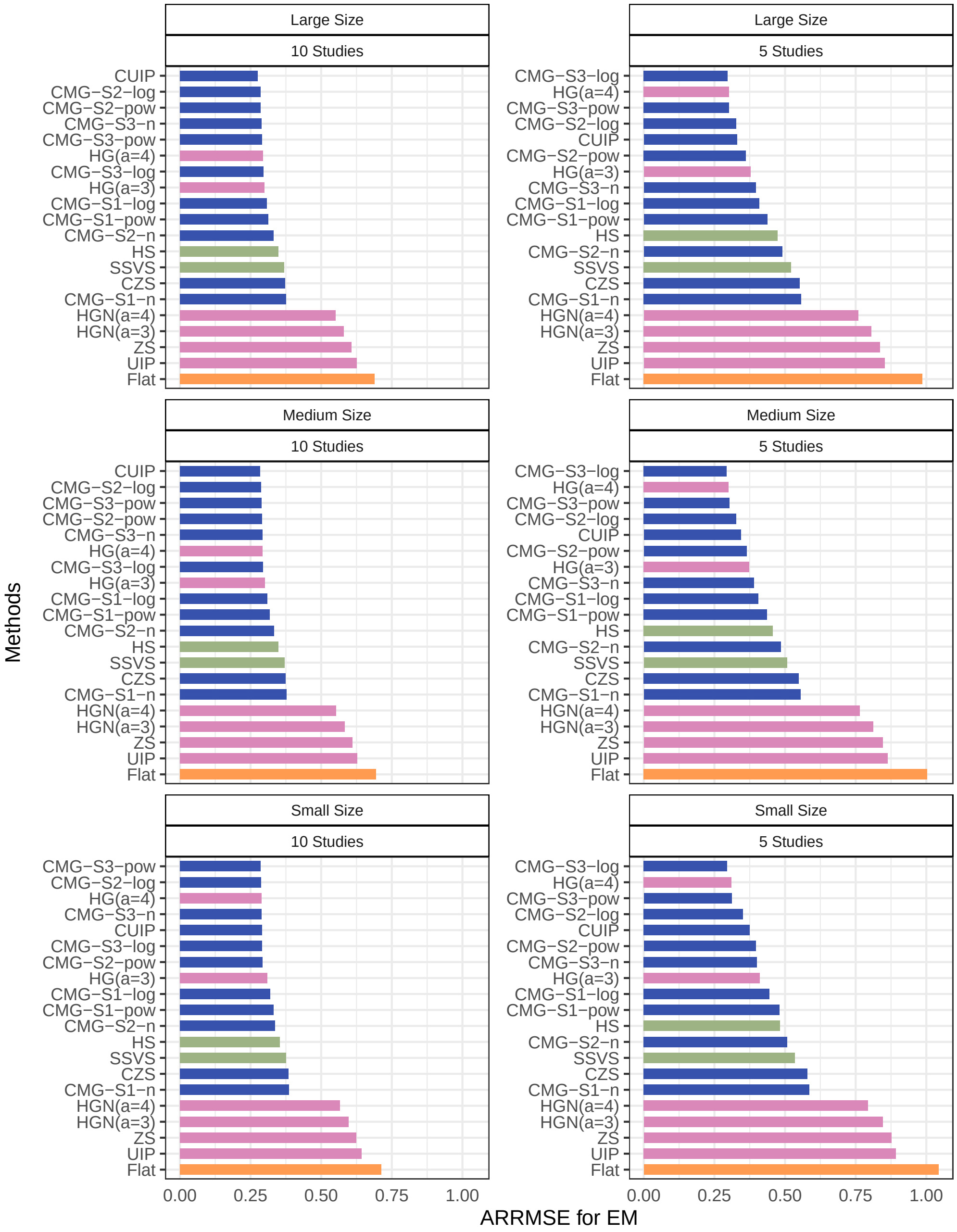

Figure S4: ARRMSE of moderation effects (γ) across 20 methods under a combination of three study size settings (from top to bottom panels) and two settings of number of studies (left and right panels) with high model sparsity, high between-trial variability, weak moderation effects and correlated covariates. In each panel, methods are arranged in increasing order of ARRMSE from top to bottom. The blue, pink, green, and orange bars correspond to the CMG, NMG, HS and SSVS, and Flat methods, respectively.

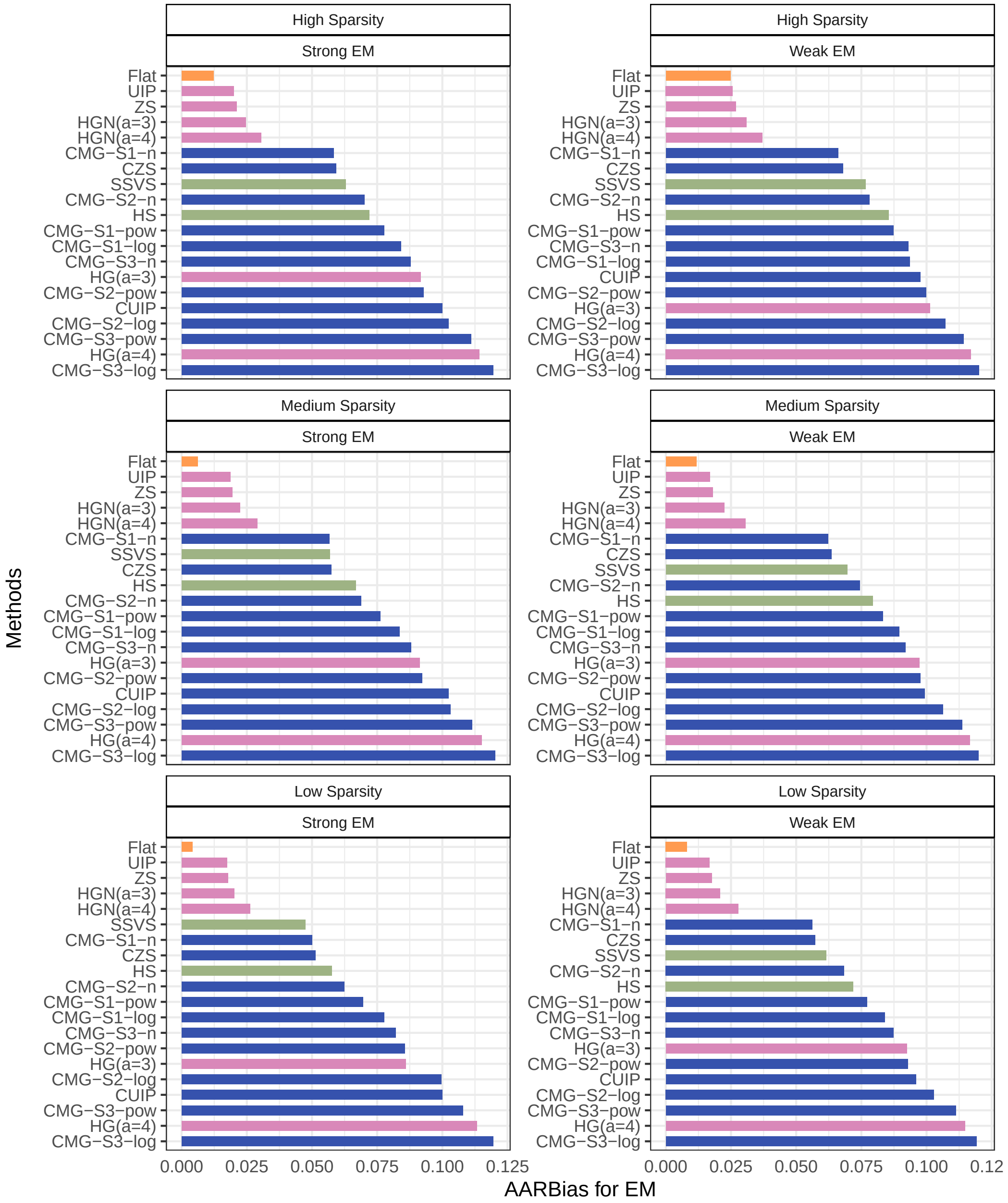

Figure S5: AARBias of moderation effects (γ) across 20 methods under a combination of three model sparsity settings (from top to bottom panels) and two magnitude of effect moderation settings (left and right panels) with high between-trial variability and correlated covariates. In each panel, methods are ordered by their performance. The blue, pink, green, and orange bars correspond to the CMG, NMG, HS and SSVS, and Flat methods, respectively.

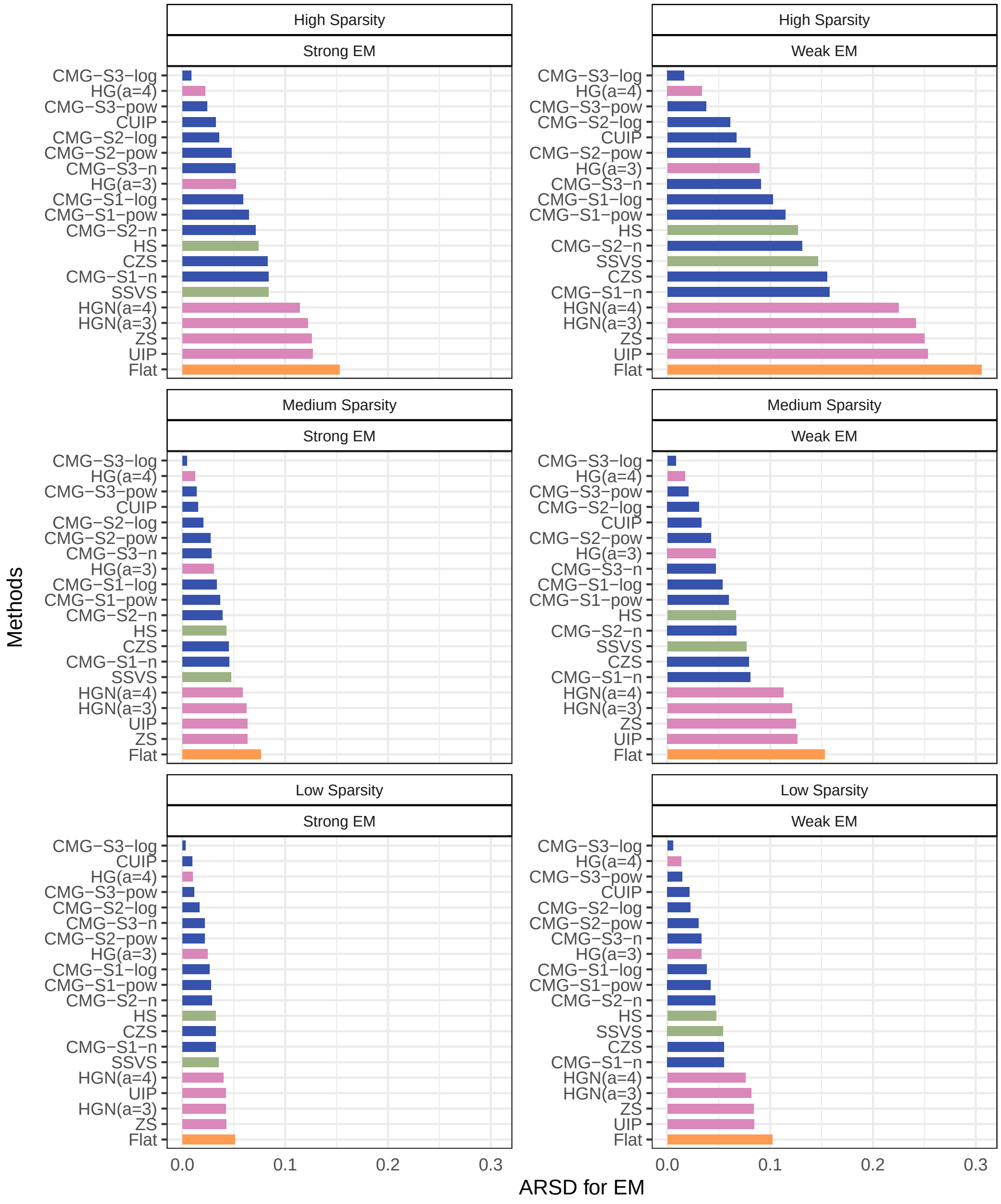

Figure S6: ARSD of moderation effects (γ) across 20 methods under a combination of three model sparsity settings (from top to bottom panels) and two magnitude of effect moderation settings (left and right panels) with high between-trial variability and correlated covariates. In each panel, methods are ordered by their performance. The blue, pink, green, and orange bars correspond to the CMG, NMG, HS and SSVS, and Flat methods, respectively.

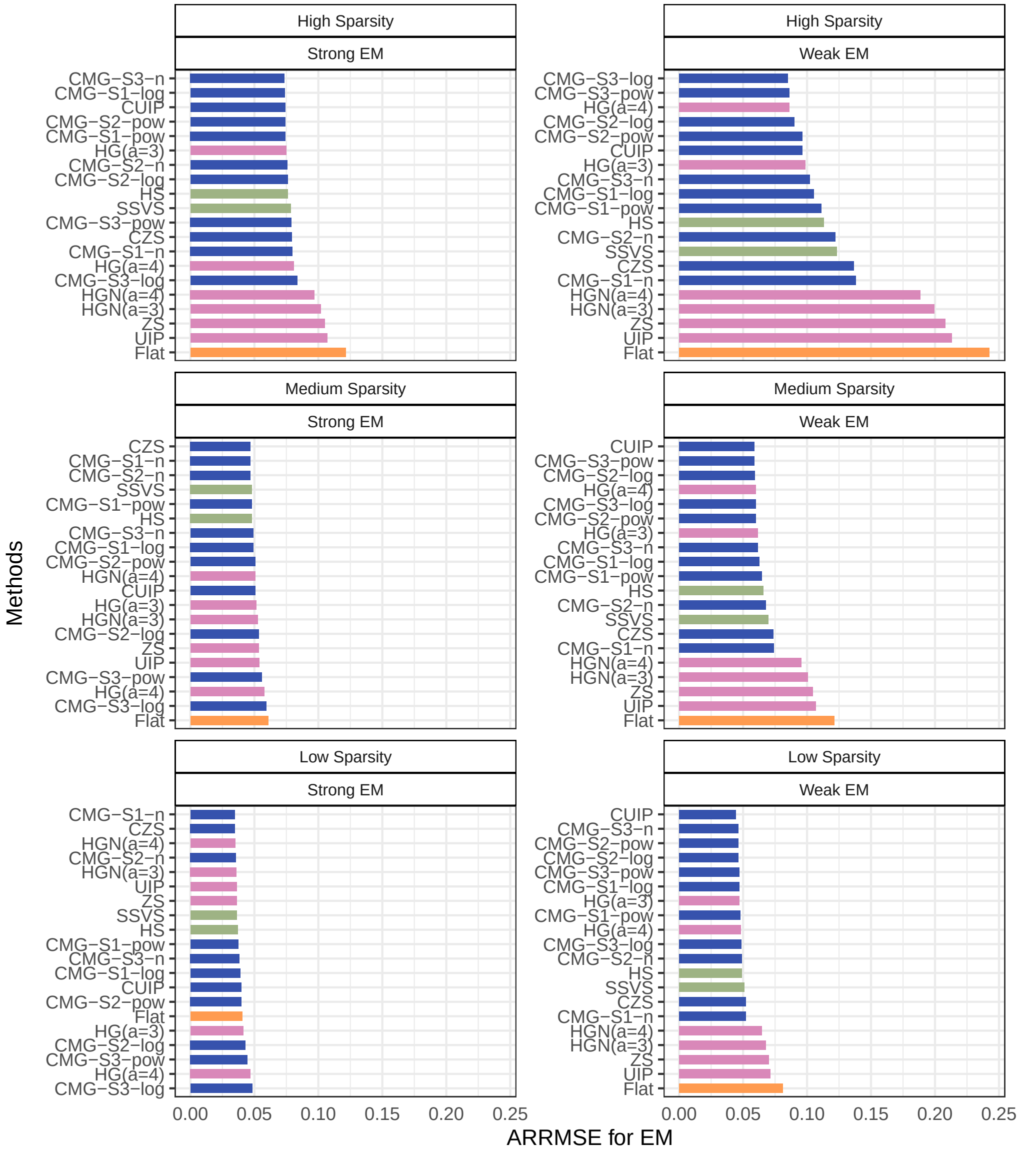

Figure S7: ARRMSE of moderation effects (γ) across 20 methods under a combination of three between-trial variability settings (from top to bottom panels) and two magnitude of effect moderation settings (left and right panels) with high model sparsity and uncorrelated covariates. In each panel, methods are ordered by their performance. The blue, pink, green, and orange bars correspond to the CMG, NMG, HS and SSVS, and Flat methods, respectively.

Section S3 Complementary Results in Real Data Analysis

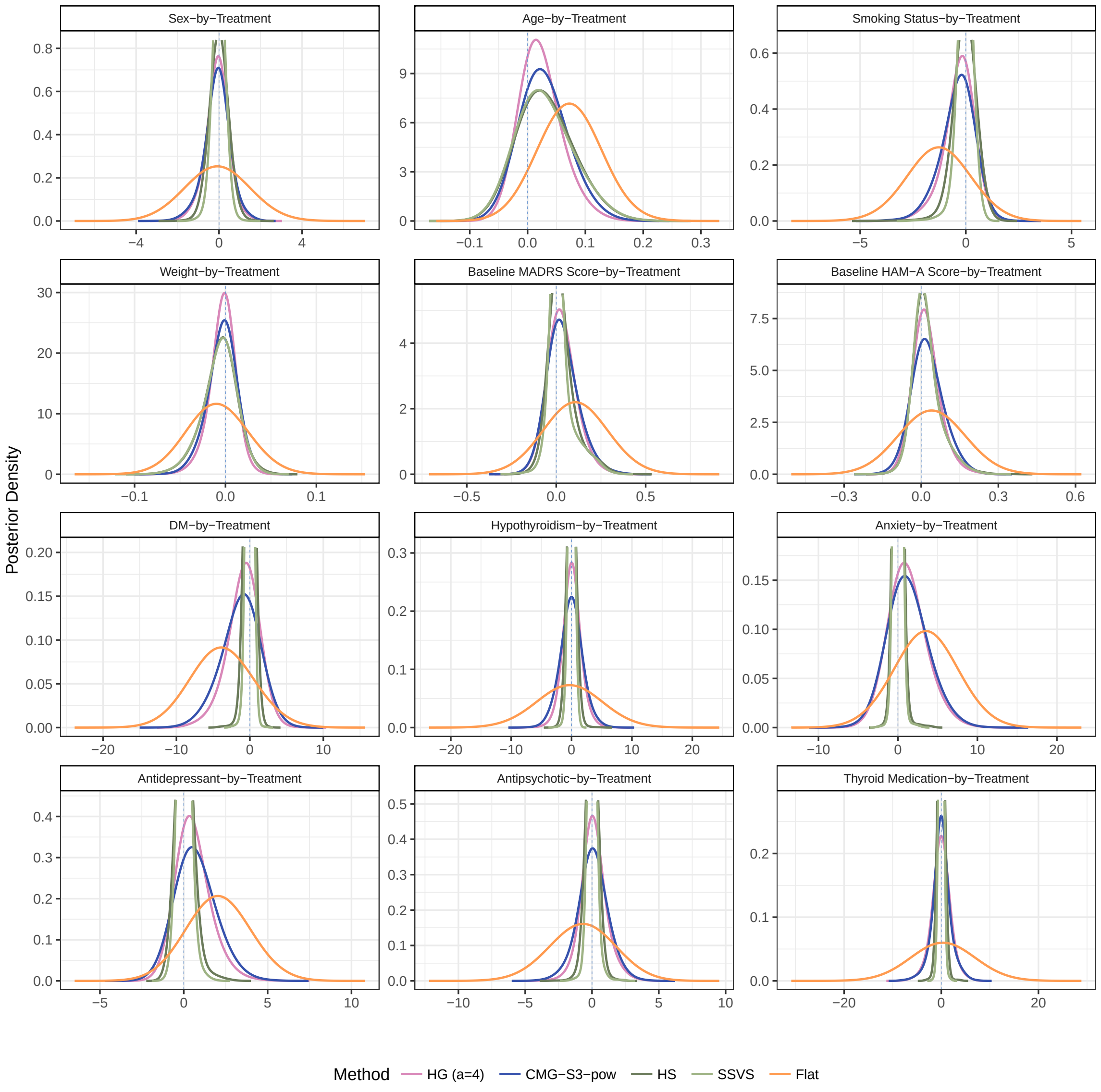

Figure S8: Posterior density plots for moderation effects (covariate-by-treatment interactions $\gamma_1, \cdots, \gamma_{12}$) under the HG (a=4) (pink), CMG-S3-pow (blue), HS(dark green), SSVS (green) and Flat (orange) methods from the IPD-MA real data analysis. For visual comparability across panels, the upper portions of the HS and SSVS density curves are truncated when sharp peaks near zero exceed the panel-specific plotting range. Accordingly, open tops in the HS and SSVS curves indicate display truncation only and do not alter the underlying posterior distributions.

Table S1: Posterior means (posterior SDs) and 95% CIs of selected model coefficients (α, $\boldsymbol{\gamma}$ and τ_α^2) estimated using the HG (a=4), CMG-S3-pow, HS, SSVS and Flat methods from the IPD-MA real data analysis.

Selected Coefficients	HG(a=4)	CMG-S3-pow	HS	SSVS	Flat
α: **Conditional Treatment Effect**	2.55 (0.85)	2.56 (0.93)	2.60 (0.82)	2.60 (0.87)	2.64 (1.08)
	(0.93, 4.20)	(0.82, 4.29)	(1.12, 4.28)	(0.73, 4.30)	(0.44, 4.63)
γ_1: **Sex-by-Treatment**	-0.08 (0.49)	-0.08 (0.53)	-0.01 (0.20)	-0.02 (0.23)	-0.04 (1.08)
	(-1.19, 0.91)	(-1.35, 1.02)	(-0.45, 0.34)	(-0.55, 0.46)	(-2.12, 2.05)
γ_2: **Age-by-Treatment**	0.02 (0.03)	0.03 (0.03)	0.03 (0.04)	0.03 (0.04)	0.07 (0.04)
	(-0.01, 0.09)	(-0.02, 0.10)	(-0.01, 0.12)	(-0.02, 0.11)	(-0.00, 0.15)
γ_3: **Smoking Status-by-Treatment**	-0.34 (0.63)	-0.39 (0.67)	-0.06 (0.30)	-0.07 (0.29)	-1.28 (1.07)
	(-1.97, 0.61)	(-1.93, 0.66)	(-0.86, 0.28)	(-0.93, 0.22)	(-3.42, 0.78)
γ_4: **Weight-by-Treatment**	-0.00 (0.01)	-0.00 (0.01)	-0.01 (0.02)	-0.01 (0.02)	-0.01 (0.02)
	(-0.03, 0.02)	(-0.03, 0.02)	(-0.05, 0.02)	(-0.05, 0.02)	(-0.06, 0.04)
γ_5: **Baseline MADRS Score-by-Treatment**	0.03 (0.07)	0.04 (0.07)	0.03 (0.07)	0.03 (0.07)	0.11 (0.13)
	(-0.08, 0.20)	(-0.09, 0.21)	(-0.07, 0.23)	(-0.08, 0.23)	(-0.14, 0.37)
γ_6: **Baseline HAM-A Score-by-Treatment**	0.02 (0.05)	0.02 (0.05)	0.02 (0.05)	0.03 (0.05)	0.04 (0.09)
	(-0.06, 0.13)	(-0.06, 0.14)	(-0.06, 0.15)	(-0.05, 0.16)	(-0.14, 0.22)
γ_7: **DM-by-Treatment**	-0.95 (1.78)	-1.24 (2.03)	-0.13 (0.72)	-0.04 (0.30)	-3.78 (3.01)
	(-5.26, 1.96)	(-6.07, 2.09)	(-2.21, 0.55)	(-0.77, 0.42)	(-9.56, 2.36)
γ_8: **Hypothyroidism-by-Treatment**	0.03 (1.27)	-0.00 (1.48)	0.01 (0.44)	-0.02 (0.27)	-0.45 (3.92)
	(-2.47, 2.77)	(-3.12, 3.20)	(-0.57, 0.69)	(-0.65, 0.44)	(-8.14, 7.36)
γ_9: **Anxiety-by-Treatment**	1.15 (1.86)	1.25 (1.97)	0.02 (0.28)	0.03 (0.33)	3.54 (2.88)
	(-1.93, 5.60)	(-1.86, 5.91)	(-0.35, 0.59)	(-0.50, 0.62)	(-2.17, 9.19)
γ_{10}: **Antidepressant-by-Treatment**	0.59 (0.83)	0.69 (0.89)	0.10 (0.38)	0.06 (0.29)	2.04 (1.33)
	(-0.58, 2.64)	(-0.61, 2.76)	(-0.21, 1.37)	(-0.26, 0.92)	(-0.61, 4.70)
γ_{11}: **Antipsychotic-by-Treatment**	0.10 (0.80)	0.08 (0.90)	0.01 (0.28)	0.02 (0.24)	-0.73 (1.72)
	(-1.52, 1.94)	(-1.95, 2.06)	(-0.54, 0.58)	(-0.42, 0.64)	(-4.11, 2.61)
γ_{12}: **Thyroid Medication-by-Treatment**	0.05 (1.62)	0.06 (1.77)	0.01 (0.33)	-0.01 (0.27)	0.51 (4.85)
	(-3.46, 3.41)	(-3.74, 4.05)	(-0.56, 0.65)	(-0.62, 0.55)	(-8.91, 9.76)
τ_α^2: **Between-trial Variability of Treatment**	1.06 (0.83)	1.09 (0.89)	1.02 (0.83)	1.06 (0.86)	1.34 (1.01)
	(0.05, 3.10)	(0.07, 3.32)	(0.05, 2.97)	(0.06, 3.23)	(0.07, 3.86)

Table S2: The scaled neighborhood criterion based posterior probabilities p_{γ_k} for covariate-by-treatment interaction ($\gamma_1, \cdots, \gamma_{12}$) using the HG ($a$=4), CMG-S3-pow, HS, SSVS and Flat methods from the IPD-MA real data analysis.

Covariate-by-Treatment Coefficients	HG (a=4)	CMG-S3-pow	HS	SSVS	Flat
γ_1: **Sex-by-Treatment**	0.76	0.75	0.88	0.88	0.68
γ_2: **Age-by-Treatment**	0.63	0.58	0.58	0.60	0.20
γ_3: **Smoking Status-by-Treatment**	0.74	0.70	0.90	0.88	0.58
γ_4: **Weight-by-Treatment**	0.74	0.73	0.73	0.72	0.66
γ_5: **Baseline MADRS Score-by-Treatment**	0.73	0.71	0.78	0.78	0.53
γ_6: **Baseline HAM-A Score-by-Treatment**	0.74	0.72	0.75	0.75	0.64
γ_7: **DM-by-Treatment**	0.71	0.69	0.92	0.89	0.38
γ_8: **Hypothyroidism-by-Treatment**	0.76	0.74	0.92	0.89	0.68
γ_9: **Anxiety-by-Treatment**	0.68	0.67	0.91	0.90	0.40
γ_{10}: **Antidepressant-by-Treatment**	0.69	0.64	0.91	0.88	0.30
γ_{11}: **Antipsychotic-by-Treatment**	0.77	0.74	0.90	0.87	0.65
γ_{12}: **Thyroid Medication-by-Treatment**	0.76	0.77	0.91	0.88	0.68
